# Nanomedicines as Guardians of the Heart: Unleashing the Power of Antioxidants to Alleviate Myocardial Ischemic Injury

**DOI:** 10.7150/thno.99961

**Published:** 2024-08-26

**Authors:** Dongjian Han, Fuhang Wang, Deliang Shen

**Affiliations:** 1 Department of Cardiology, The First Affiliated Hospital of Zhengzhou University, Zhengzhou, 450052, China.; 2 Key Laboratory of Cardiac Injury and Repair of Henan Province, Zhengzhou, China.

**Keywords:** myocardial ischemic injury, oxidative stress, antioxidant therapy, nanomedicines, drug delivery systems

## Abstract

Ischemic heart disease (IHD) is increasingly recognized as a significant cardiovascular disease with a growing global incidence. Interventions targeting the oxidative microenvironment have long been pivotal in therapeutic strategies. However, many antioxidant drugs face limitations due to pharmacokinetic and delivery challenges, such as short half-life, poor stability, low bioavailability, and significant side effects. Fortunately, nanotherapies exhibit considerable potential in addressing IHD. Nanomedicines offer advantages such as passive/active targeting, prolonged circulation time, enhanced bioavailability, and diverse carrier options. This comprehensive review explores the advancements in nanomedicines for mitigating IHD through oxidative stress regulation, providing an extensive overview for researchers in the field of antioxidant nanomedicines. By inspiring further research, this study aims to accelerate the development of novel therapies for myocardial injury.

## 1. Introduction

Ischemic heart disease (IHD), a prevalent cardiovascular disease, often leads to chronic heart failure (HF) and significantly increases global morbidity and mortality 1. In 2017, the World Health Organization (WHO) reported approximately 8.9 million deaths associated with IHD, with an increasing trend in annual incidence 2. Myocardial infarction (MI) is a severe cardiac event triggered by coronary artery blockage, restricting blood flow and leading to prolonged ischemic damage 3. Timely reperfusion, achieved via thrombolysis or primary percutaneous coronary intervention (PCI), is critical for mitigating ischemic damage and reducing the infarct size. However, this reperfusion can also provoke myocardial ischemia/reperfusion injury (MI/RI), resulting in further tissue damage and increased cardiomyocyte (CM) apoptosis 4. As prominent diseases within IHD, MI and MI/RI necessitate ongoing research into their molecular and cellular pathways. This research is crucial for the development of targeted therapies and for deepening our understanding of these conditions.

Oxidative stress, characterized by an excess of reactive oxygen species (ROS) that overwhelms the body's antioxidant defenses, plays a pivotal role in the progression of IHD by causing oxidative damage to cellular components and exacerbating tissue injury 5. An imbalance between elevated ROS production and a diminished antioxidant response leads to significant oxidative damage and triggers pro-inflammatory pathways, ultimately causing CM apoptosis and tissue necrosis 6. Therefore, strategies to mitigate oxidative stress are crucial for improving cardiac function and treating IHD. Various drugs, such as scutellarin 7, adenosine 8, bakuchiol 9, and melatonin (Mel) 10, have shown promise in alleviating IHD. Despite their efficacy, these agents face challenges like poor targeting and adverse effects, highlighting the need for more effective and safer therapeutic options 11.

In recent years, nanomedicine has significantly advanced, marked by an increase in research involving preclinical and clinical trials. These nanomedicines are often designed by encapsulating therapeutic agents within various carriers, enhancing therapeutic effectiveness and safety compared to free drugs 12. The customization of nanomedicines involves careful consideration of properties such as drug load, dimensions, morphology, surface charge, and targeting moieties to achieve the desired therapeutic effects 13. This customization aims to optimize pharmacokinetics and pharmacodynamics, control drug release, enhance targeted delivery, prolong circulation in blood, minimize side effects, improve biocompatibility, and increase tissue penetration 14. A wide range of inorganic and organic materials, including liposomes, polymeric NPs (PNPs), and extracellular vesicles (EVs), have been explored as carriers 15. Notably, the development of diagnostic, therapeutic, and theranostic nanomedicines facilitates early detection and precise treatment of IHD.

**Figure 1:**
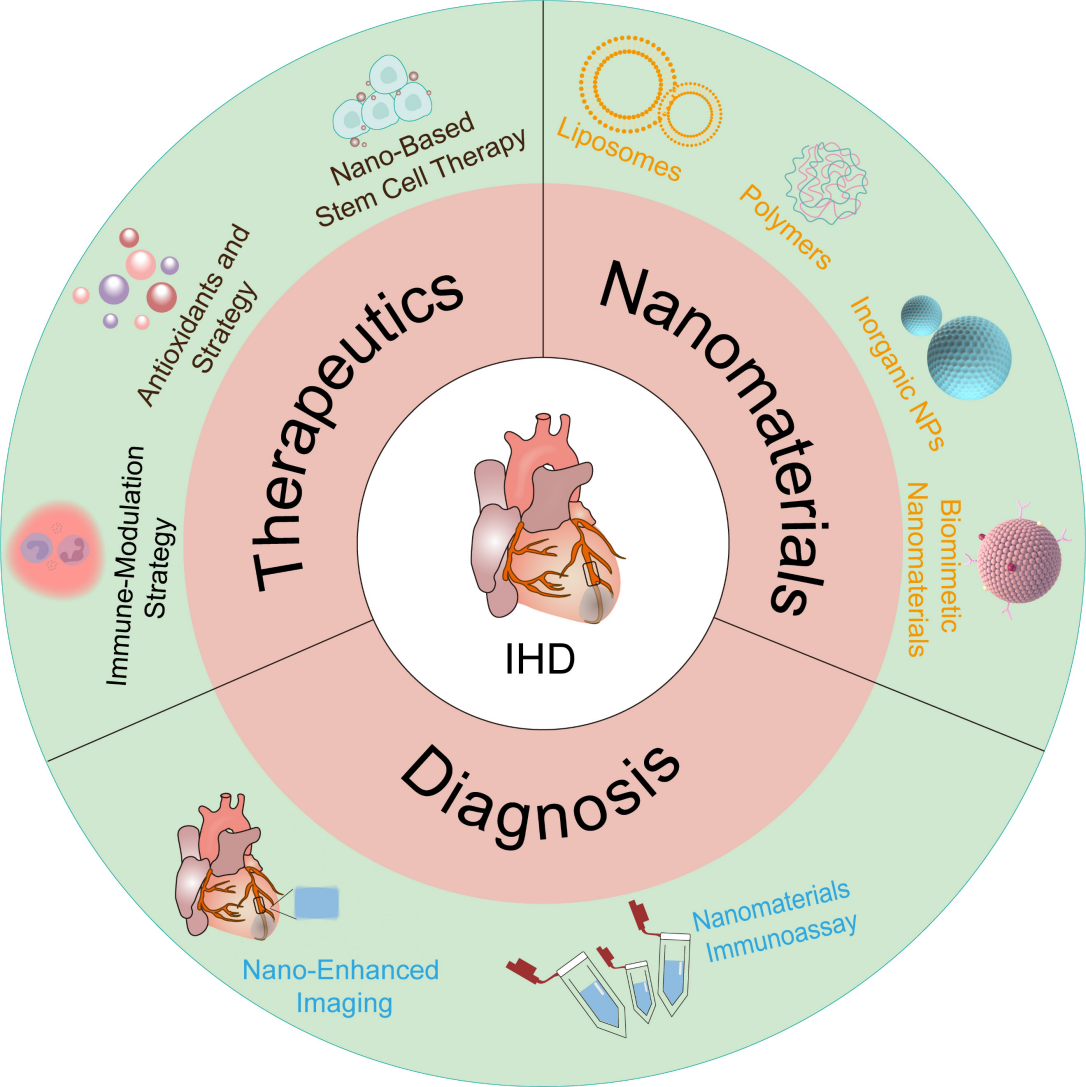
The groundbreaking nanomaterial schematics for precise IHD therapy and diagnosis.

Recently, there has been a surge in interest in using nanomedicines for diagnosing and treating IHD (Figure 1). This review not only summarizes current advancements but also provides new insights into optimizing nanomedicine applications for IHD treatment, identifying key areas where future research can bridge existing gaps.

## 2. Pathological mechanisms of IHD

Coronary atherosclerosis, a common cardiovascular disease, results from the deposition of fatty substances and cholesterol in coronary arteries, leading to obstructive plaques 16. The severity of this condition is determined by the degree of arterial occlusion and its impact on blood flow 17. Risk factors include age, sex, genetic factors, lifestyle choices such as smoking, poor diet, lack of exercise, as well as conditions like hypertension, diabetes, and dyslipidemia 18. As a significant global health concern, coronary atherosclerosis is a leading cause of death and illness worldwide 19.

MI, commonly known as a heart attack, is a critical medical emergency caused by the cessation of blood flow to the heart muscle, resulting in tissue damage or death 20. The sudden arterial occlusion is typically caused by the rupture of atherosclerotic plaques, which activates the coagulation system 21. Plaque rupture releases pro-coagulant substances, activating thrombin and platelets and leading to thrombus formation at the site of the plaque, which can obstruct or even completely block the affected vessels 22, 23. Reperfusion therapy aims to restore blood flow to the ischemic myocardium, effectively reducing ischemic damage and minimizing the infarct size. However, reperfusion can also cause MI/RI, leading to further cardiac damage and potentially reducing the success of revascularization 24. Understanding the pathophysiological mechanisms of IHD is crucial for targeted therapeutic interventions.

**Figure 2:**
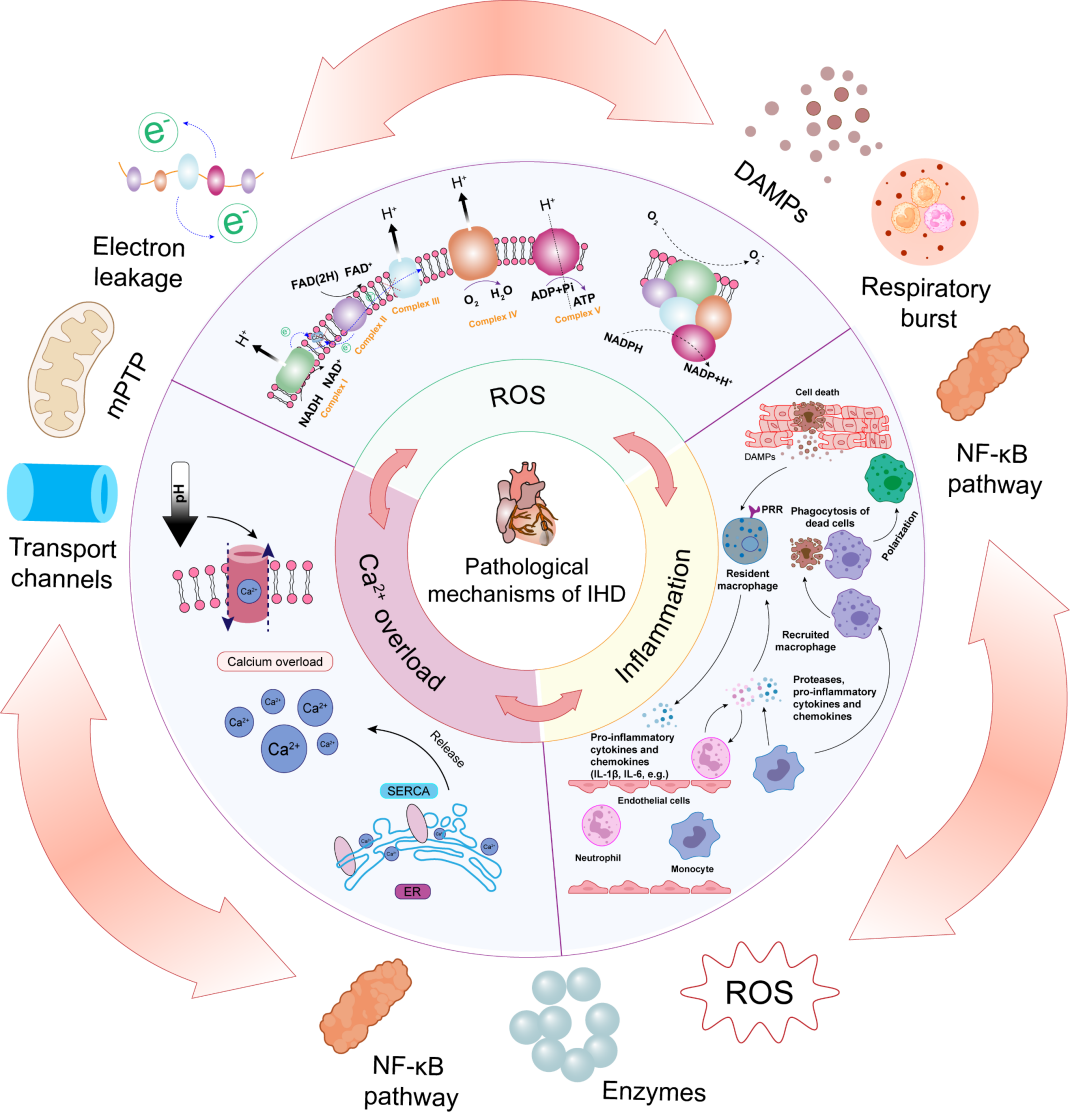
A schematic diagram of the mechanisms causing IHD, including oxidative stress, inflammation, and calcium overload.

This section reviews the complex mechanisms of IHD, characterized by a complex interplay of pathophysiological processes, including inflammation, Ca^2+^ overload, and notably, oxidative stress (Figure 2). The intricate interaction and convergence of these factors underscore the need for a comprehensive understanding of their mechanisms, which is essential for developing effective therapeutic strategies for IHD.

### 2.1. Oxidative and nitrative stress

ROS are integral to the pathophysiology of IHD, functioning as both signaling molecules and mediators of cellular damage. The generation of ROS during these events is a complex, multi-step process that is tightly linked to the metabolic disturbances and bioenergetic crises that characterize ischemia and reperfusion 25. A comprehensive understanding of the pathways involved in ROS production and their subsequent pathological roles is essential for developing targeted therapeutic strategies aimed at mitigating myocardial injury.

The mitochondrial electron transport chain (ETC) is the primary source of ROS during MI and MI/RI. Under physiological conditions, electrons from NADH and FADH2 are sequentially transferred through complexes I to IV of the ETC, culminating in the reduction of oxygen to water at complex IV. However, ischemia results in a sharp decline in oxygen availability, leading to a backlog of reduced electron carriers and excessive reduction of ETC components. Upon reperfusion, the sudden influx of oxygen reignites the ETC, causing excessive flux of electrons. This hyperactive state of the ETC results in substantial electron leakage at complexes I and III, where electrons prematurely reduce molecular oxygen to form superoxide anions (•O_2_^-^) 26.

The formation of superoxide marks the initiation of a cascading sequence of ROS generation. Superoxide dismutase (SOD) rapidly converts superoxide into hydrogen peroxide (H_2_O_2_), which, although less reactive, can freely diffuse across membranes and contribute to oxidative stress 27. In the presence of transition metals such as iron, H_2_O_2_ is converted into hydroxyl radicals (•OH) via Fenton chemistry 28. •OH, due to their high reactivity, inflict severe damage on lipids, proteins, and DNA, thereby compromising the structural and functional integrity of myocardial cells.

Beyond mitochondrial sources, NADPH oxidase (NOX) enzymes represent a significant non-mitochondrial pathway for ROS production in IHD. NOX enzymes are distinct in that their primary function is the deliberate generation of ROS. Upon activation by ischemic stress, inflammatory cytokines, or mechanical strain, NOX enzymes catalyze the transfer of electrons from NADPH to molecular oxygen, producing superoxide 29. Among the NOX isoforms, NOX2 and NOX4 are particularly implicated in cardiac injury, with NOX2 contributing to the acute ROS burst during reperfusion, and NOX4 being associated with sustained ROS production and the promotion of fibrotic remodeling. The ROS generated by NOX enzymes are not merely byproducts of cellular stress; they also act as critical modulators of redox-sensitive signaling pathways. These ROS can activate a range of downstream kinases and transcription factors, such as protein kinase C (PKC) and nuclear factor-kappa B (NF-κB), which amplify inflammatory responses, induce apoptosis, and disrupt metabolic homeostasis, all of which contribute to the exacerbation of myocardial damage in IHD 30.

A less conventional but highly relevant source of ROS in the setting of IHD is the uncoupling of endothelial nitric oxide synthase (eNOS). Under normal conditions, eNOS generates nitric oxide (NO), a molecule with potent vasodilatory and cytoprotective effects. However, under oxidative stress, such as that encountered during reperfusion, eNOS can become uncoupled due to the oxidation of its cofactor tetrahydrobiopterin (BH4) or a deficiency in L-arginine. When uncoupled, eNOS shifts from producing NO to generating superoxide, which not only exacerbates oxidative stress but also diminishes NO availability, leading to endothelial dysfunction and impaired vasodilation 31. This dual effect further perpetuates ischemic injury by impairing perfusion and increasing the oxidative burden within the myocardium.

### 2.2 Inflammation

Inflammation is a fundamental response to IHD, playing a crucial role in both the progression of acute myocardial damage and the subsequent remodeling of cardiac tissue. This complex inflammatory process is rapidly initiated following ischemia and further intensified upon reperfusion, driven by the activation of resident immune cells, the recruitment of circulating leukocytes, and the release of pro-inflammatory mediators 32. Understanding the pathways and mechanisms by which inflammation is generated and perpetuated is essential for elucidating the full spectrum of myocardial injury in these conditions.

The inflammatory cascade in IHD is triggered by the release of damage-associated molecular patterns (DAMPs) from necrotic CMs and stressed cells. These DAMPs, which include high-mobility group box 1 (HMGB1), heat shock proteins, and extracellular matrix degradation products, are recognized by pattern recognition receptors (PRRs) such as Toll-like receptors (TLRs) and nucleotide-binding oligomerization domain-like receptors (NLRs) on resident immune cells, including macrophages and dendritic cells 33. The engagement of these receptors activates intracellular signaling cascades, notably the NF-κB and mitogen-activated protein kinase (MAPK) pathways, culminating in the transcriptional upregulation of pro-inflammatory cytokines like tumor necrosis factor-alpha (TNF-α), interleukin-1 beta (IL-1β), and interleukin-6 (IL-6) 34.

These cytokines orchestrate a robust inflammatory response, promoting the recruitment of neutrophils and monocytes to the ischemic myocardium. Neutrophils are among the first responders, infiltrating the myocardium within hours of reperfusion. These cells release a variety of enzymes, ROS, and cytokines that exacerbate myocardial injury, not only through direct cellular damage but also by amplifying the inflammatory milieu. Monocytes, which differentiate into macrophages upon tissue entry, sustain the inflammatory response and initiate the clearance of dead cells and debris through phagocytosis 32. Macrophages also secrete growth factors that are essential for tissue repair, yet their prolonged activation can lead to excessive inflammation and fibrosis, contributing to adverse cardiac remodeling.

A critical aspect of this inflammatory response is the polarization of macrophages within the heart. Macrophages exhibit different phenotypes, notably the pro-inflammatory M1 type and the anti-inflammatory M2 type. The transition from M1 to M2 macrophages is essential for resolving inflammation and promoting tissue repair 35. The process of macrophage polarization is highly dynamic and influenced by various microenvironmental factors, including the presence of cytokines, growth factors, and metabolic by-products. The interplay between oxidative stress and macrophage phenotype suggests that a nuanced understanding of the redox environment within ischemic tissue could lead to more targeted and effective therapeutic strategies. Antioxidant nanomedicines, by modulating this redox balance, hold the potential not only to shift macrophages toward the M2 phenotype but also to create a microenvironment that favors long-term cardiac repair and remodeling 36.

This persistent and intense innate immune response not only leads to the necrosis and apoptosis of CMs but also exacerbates myocardial damage and diminishes the effectiveness of treatments 37. By promoting M2 macrophage polarization, antioxidant nanomedicines help modulate the inflammatory microenvironment, mitigating myocardial damage and supporting cardiac repair. For example, antioxidant nanomedicines have been shown to enhance M2 macrophage polarization, improving cardiac function and reducing infarct size in preclinical models of MI 35. The challenge of modulating the inflammatory microenvironment lies in the precise control of the timing and extent of M2 macrophage activation. Overactive M2 polarization may inadvertently lead to fibrosis, while insufficient M2 activity could prolong inflammation and hinder repair 38. Therefore, future research should focus on fine-tuning antioxidant nanomedicine formulations to achieve an optimal balance, potentially through the use of stimuli-responsive systems that release therapeutic agents in response to specific inflammatory signals.

Inflammatory processes significantly boost ROS production through several mechanisms. During IHD, inflammatory cytokines such as TNF-α, IL-1β, and IL-6 activate NOX, particularly NOX2 and NOX4, in neutrophils, macrophages, and endothelial cells. These enzymes transfer electrons from NADPH to oxygen, directly generating superoxide, a primary ROS. Moreover, the activation of NF-κB and MAPK pathways by these cytokines upregulates NOX enzymes and other ROS-generating systems, further amplifying ROS production 39.

ROS, in turn, act as potent amplifiers of the inflammatory response. ROS activate redox-sensitive transcription factors like NF-κB and activator protein-1 (AP-1), leading to the transcription of pro-inflammatory genes. This results in increased production of cytokines and chemokines, which recruit more immune cells to the injured myocardium, thereby sustaining and intensifying inflammation 40. Additionally, cause oxidative damage to cellular components, leading to the release of further DAMPs, which activate PRRs on immune cells, perpetuating the cycle of inflammation and ROS generation 39.

In summary, inflammation and ROS generation are mutually reinforcing processes in IHD. Inflammatory signaling upregulates ROS production, while ROS further intensify inflammation by activating key signaling pathways and causing oxidative damage, thus creating a self-perpetuating cycle that exacerbates myocardial injury.

### 2.3 Ca^2+^ overload

Ischemic insult precipitates a cascade of metabolic disturbances, with the initial deprivation of oxygen halting oxidative phosphorylation in mitochondria. This disruption leads to the collapse of mitochondrial membrane potential and a precipitous drop in ATP production, compelling the cell to rely on anaerobic glycolysis. The accumulation of lactic acid from glycolysis induces intracellular acidosis, which rapidly reverses the reactivation of Na^+^-Ca^2+^ exchange channels, leading to a substantial influx of Ca^2+^ into the cells and an increase in intracellular Ca^2+^ concentrations 41. Therefore, reperfusion is considered a significant factor in exacerbating Ca^2+^ overload.

Elevated intramitochondrial calcium concentrations stimulate key dehydrogenases within the tricarboxylic acid (TCA) cycle, such as isocitrate dehydrogenase and α-ketoglutarate dehydrogenase. This stimulation leads to an overproduction of NADH and FADH₂, which feed into the ETC. When the ETC becomes excessively loaded with electrons beyond its oxidative capacity, electron leakage occurs, particularly at complexes I and III. These leaked electrons prematurely react with molecular oxygen, resulting in the formation of superoxide, a primary form of ROS. This calcium-induced overproduction of superoxide within mitochondria is a significant contributor to cellular oxidative stress and subsequent tissue injury 42.

In addition, calcium overload contributes to mitochondrial membrane potential depolarization, which can trigger the opening of the mitochondrial permeability transition pore (mPTP). The opening of the mPTP leads to a loss of the mitochondrial membrane potential, disrupting the proton gradient essential for ATP synthesis and causing a cessation of oxidative phosphorylation. This disruption not only diminishes the cell's energy supply but also precipitates further ROS generation, as the electron transport chain becomes dysregulated. The resultant ROS production exacerbates mitochondrial dysfunction, creating a feed-forward loop where calcium overload and ROS reinforce each other's pathological effects 43.

ROS can initiate protein denaturation, enzyme inactivation, and peroxidation of polyunsaturated fatty acids in cell membranes, thereby disrupting membrane permeability. ROS directly attack cellular components such as ion transport channels, sarcoplasm, and mitochondrial supercomplexes, leading to cellular Ca^2+^ overload. Moreover, ROS-induced oxidative stress exerts a profound effect on calcium signaling. Oxidative modifications of calcium-handling proteins, including the ryanodine receptors and sarcoplasmic reticulum (SR) Ca²⁺ ATPase, disrupt their normal function, exacerbating calcium mishandling and amplifying Ca²⁺ overload 44. The pathological interplay between ROS and Ca²⁺ overload thus constitutes a vicious cycle, with each amplifying the other through a network of interrelated signaling pathways and metabolic disturbances. This cycle not only drives the acute phase of myocardial injury but also sets the stage for chronic pathological remodeling and HF.

Additionally, elevated intracellular Ca^2+^ levels can activate NF-κB, a key transcription factor that regulates the expression of pro-inflammatory cytokines such as TNF-α, IL-1β, and IL-6. Moreover, Ca^2+^ overload can activate inflammasomes, particularly the NLRP3 inflammasome, which further amplifies the inflammatory response by promoting the maturation and release of IL-1β and interleukin-18 (IL-18). Conversely, inflammation exacerbates calcium overload through multiple mechanisms 45. Pro-inflammatory cytokines can impair calcium homeostasis by downregulating the expression and function of calcium-handling proteins, such as the SR Ca²⁺-ATPase (SERCA) and plasma membrane Ca²⁺-ATPase (PMCA) 46. Inflammatory mediators also increase oxidative stress, which disrupts calcium channels and transporters, leading to further calcium influx and overload 39.

In IHD, a self-reinforcing cycle exists between oxidative stress, inflammation, and calcium overload. ROS generated during these events amplify inflammation by activating redox-sensitive pathways, leading to the release of pro-inflammatory cytokines and further ROS production. In turn, inflammation exacerbates calcium overload by disrupting calcium-handling proteins and increasing oxidative stress. Calcium overload contributes to additional ROS generation through mitochondrial dysfunction and activation of inflammasomes, which further intensifies the inflammatory response. This vicious cycle not only exacerbates acute myocardial damage but also drives long-term pathological remodeling, highlighting the need for targeted therapies that interrupt these interconnected processes.

## 3. Antioxidants

In exploring the cellular redox balance, antioxidants are hypothesized to function via multiple mechanisms: (a) scavenging ROS or their precursors, (b) inhibiting ROS production, (c) binding metal ions to reduce ROS catalysis, (d) enhancing the endogenous synthesis of antioxidants, and (e) upregulating antiapoptotic genes such as Bcl-2 to mitigate cell death. Antioxidants are categorized as endogenous (synthesized within the body) or exogenous (acquired externally). These compounds display a spectrum of properties that enable them to mitigate oxidative damage and forestall the development of various diseases through a complex network of cellular defense mechanisms. Understanding their mechanisms is crucial for developing nanomedicine interventions for IHD.

### 3.1 Endogenous antioxidants

Endogenous antioxidants, including enzymatic antioxidants such as GPx, SOD, GSH, and catalase (CAT) and nonenzymatic antioxidants such as bilirubin, melanin, Mel, nucleic acids (NAs), and various gases, play vital roles in defending against oxidative stress in IHD. Both enzymatic and nonenzymatic antioxidant systems collaboratively maintain the body's redox balance and serve as direct or indirect targets for antioxidant interventions.

#### 3.1.1 Enzymatic antioxidants

SOD, CAT, and GPx, along with GSH, are pivotal endogenous antioxidants. SOD, a potent enzymatic protector against oxidative stress, catalyzes the conversion of •O_2_^-^ to H_2_O_2_, which is then reduced by CAT and GPx to prevent peroxynitrite formation 27. CAT, which is prevalent in eukaryotic cells, degrades H_2_O_2_ into water and O_2_, thus preserving cellular redox homeostasis. Thus, the combined use of SOD and CAT is considered a superior choice as it can further reduce the harmful effects of H_2_O_2_. In conjunction with GSH, GPx can catalyze the reduction of H_2_O_2_, which is crucial for maintaining cellular redox homeostasis. A deficiency in GPx increases vulnerability to I/R injuries, whereas its overexpression can prevent left ventricular remodeling and failure post-MI 47. The fundamental role of enzymatic antioxidants such as SOD, CAT, and GPx in maintaining redox homeostasis cannot be overstated. These enzymes not only mitigate oxidative stress by neutralizing ROS but also play critical roles in signaling pathways that govern cell survival, proliferation, and apoptosis. However, the therapeutic application of these enzymes faces significant challenges. The poor *in vivo* stability and bioavailability of these enzymes necessitate innovative delivery systems to enhance their therapeutic efficacy. Nanotechnology-based delivery systems, such as encapsulation within nanoparticles or conjugation with polymers, could improve the stability, bioactivity, and targeted delivery of these enzymes, potentially overcoming current limitations.

Additionally, the interplay between these enzymatic antioxidants and other cellular defense mechanisms, such as autophagy and apoptosis, warrants further exploration. Understanding how these pathways intersect could reveal new therapeutic strategies that exploit the body's natural defense systems. For instance, enhancing GPx4 activity not only prevents ferroptosis but could also be strategically targeted to modulate the overall cellular response to oxidative stress, offering a novel approach to managing conditions like MI and MI/RI 48. Furthermore, the genetic modulation of these enzymes, either through overexpression or via CRISPR/Cas9-mediated editing, offers another potential avenue for therapeutic intervention. While current research has demonstrated the benefits of enzymatic antioxidant overexpression in preclinical models, translating these findings into clinical applications remain challenging (Table 1).

#### 3.1.2 Nonenzymatic antioxidants

Nonenzymatic antioxidants, which also originate within the body, neutralize free radicals through direct reactions. For instance, bilirubin, a byproduct of heme catabolism, acts as a natural ROS scavenger, offering antioxidative properties that surpass those of vitamins E and C 49. Melanin, a polymer pigment, serves multiple functions, including radical scavenging and radiation protection. Synthetic analogs like polydopamine (PDA) are being developed to capture alkylperoxyl radicals 50. NAs also exert antioxidant effects by modulating intracellular antioxidant enzymes, though their delivery is hindered by properties like charge, size, and instability 51. Gaseous molecules, essential for cellular signaling and physiological functions, can reduce oxidative stress, and nanomedicine platforms like gas-generating nanoplatforms (GGNs) enhance their therapeutic applications 52. Coenzyme Q10 (CoQ10), a lipid-soluble benzoquinone, is effective in energy production and antioxidant functions, highlighting its potential in treating IHD 53. Mel, known for its potent antioxidant properties, shows promise in various oxidative stress models, especially due to its mitochondrial targeting ability 54.

### 3.2 Exogenous antioxidants

Exogenous antioxidants, such as vitamins, natural small molecule drugs (NSMs), and synthetic antioxidants, complement endogenous defenses by regulating oxidative balance.

#### 3.2.1 Vitamins

Exogenous antioxidants, mainly obtained through dietary intake from fruits, vegetables, nuts, and seeds, play a crucial role in neutralizing free radicals. Vitamins A, C, and E, polyphenols, and certain minerals are particularly significant among these antioxidants 55. They complement endogenous antioxidants, forming a vital defense system within the body. However, the effects of exogenous antioxidants vary under different experimental conditions. For instance, some studies have shown that antioxidant supplements can enhance endothelial function 56, especially when endogenous oxidative stress is high. Conversely, a study on normal domestic pigs indicated potential negative long-term cardiovascular effects from prolonged supplementation with vitamins E and C. This was attributed to increased oxidative stress in the arterial wall, possibly due to endothelial NO synthase uncoupling or the prooxidant effects of vitamin radicals 57. These findings highlight that the benefits of antioxidants depend on the context and duration of their use. However, the stability of vitamins can be significantly affected by physical and chemical factors like light, temperature, enzymatic oxidation, metal ions, and alkaline pH. These factors often lead to the rapid degradation of vitamins into less effective forms 58. To overcome this issue, an effective delivery system is essential.

#### 3.2.2 NSMs

Secondary metabolites, also known as NSMs, have a wide range of biological activities, primarily through interactions with biological receptors. The antioxidant properties of NSMs are chiefly derived from four structural motifs: highly conjugated hydroxyl, amino, thiol, and isoprenoid groups 59. Curcuminoids, a notable group within the NSMs family, exhibit significant therapeutic potential due to their antioxidant, anti-inflammatory, and anticarcinogenic properties. The antioxidant efficacy of these compounds is enhanced by the presence of a para-hydroxyl group on two phenyl rings, supported by electron-donating groups such as methoxy groups 60. Flavonoids, another subset of NSMs, feature a C6-C3-C6 three-ring core structure and exhibit antioxidant effects primarily through hydrogen atom transfer (HAT) from their phenolic OH groups. The number of free hydroxyl groups in flavonoids correlates with their antioxidant potency, although this relationship has certain limitations 61.

Even though these compounds hold promise, over 90% of natural compounds extracted from organisms do not advance in drug development due to poor solubility, stability, or pharmacokinetic properties 59, 62, 63. Nevertheless, the focus of pharmaceutical research is evolving toward the exploration of active phase states through non-covalent interactions, which may revolutionize drug discovery. Additionally, the continuous development of advanced delivery systems plays a crucial role in improving the pharmacodynamic characteristics of these drugs, thereby enhancing their therapeutic efficacy and bioavailability.

#### 3.2.3 Synthetic antioxidants

Natural antioxidants derived from fruits and vegetables play a crucial role in promoting health and preventing diseases. Despite their advantages, these antioxidants often face challenges such as difficult extraction and instability, limiting their industrial application 64. In the food industry, synthetic antioxidants are preferred due to their consistent availability and stability. These synthetic antioxidants benefit from diverse raw material sources, advanced manufacturing technologies, cost-effectiveness, fewer side effects, and ease of procurement 65. As a result, the food industry frequently chooses synthetic options to ensure product quality and longevity. Common synthetic phenolic antioxidants in the food sector include butylated hydroxyanisole (BHA), butylated hydroxytoluene (BHT), tertiary butylhydroquinone (TBHQ), and propyl gallate (PG). These antioxidants are crucial for inhibiting spoilage, stabilizing products, and extending the longevity of food items 66. Their use provides economic benefits and enhanced food safety, although their concentration must be carefully managed to avoid adverse health implications.

The transformation of antioxidants into diverse metabolites under specific environmental or biochemical conditions is a crucial area of study. These metabolites, which vary significantly depending on the reaction conditions and the organism involved, play integral roles in antioxidative processes. Understanding these transformation mechanisms is essential for advancing our knowledge of both natural and synthetic antioxidants. Moreover, the application of nanocarriers in the synthesis and delivery of antioxidants is pivotal. Nanocarriers enhance the stability, bioavailability, and targeted delivery of antioxidants, thereby maximizing their therapeutic efficacy. This integration of nanotechnology in antioxidant research not only facilitates the precise modulation of metabolic pathways but also addresses the limitations associated with conventional antioxidant therapies. Hence, the incorporation of nanocarriers is indispensable for the effective utilization of synthetic antioxidants in therapeutic interventions.

## 4. Application of nanocarriers in IHD therapy

**Figure 3:**
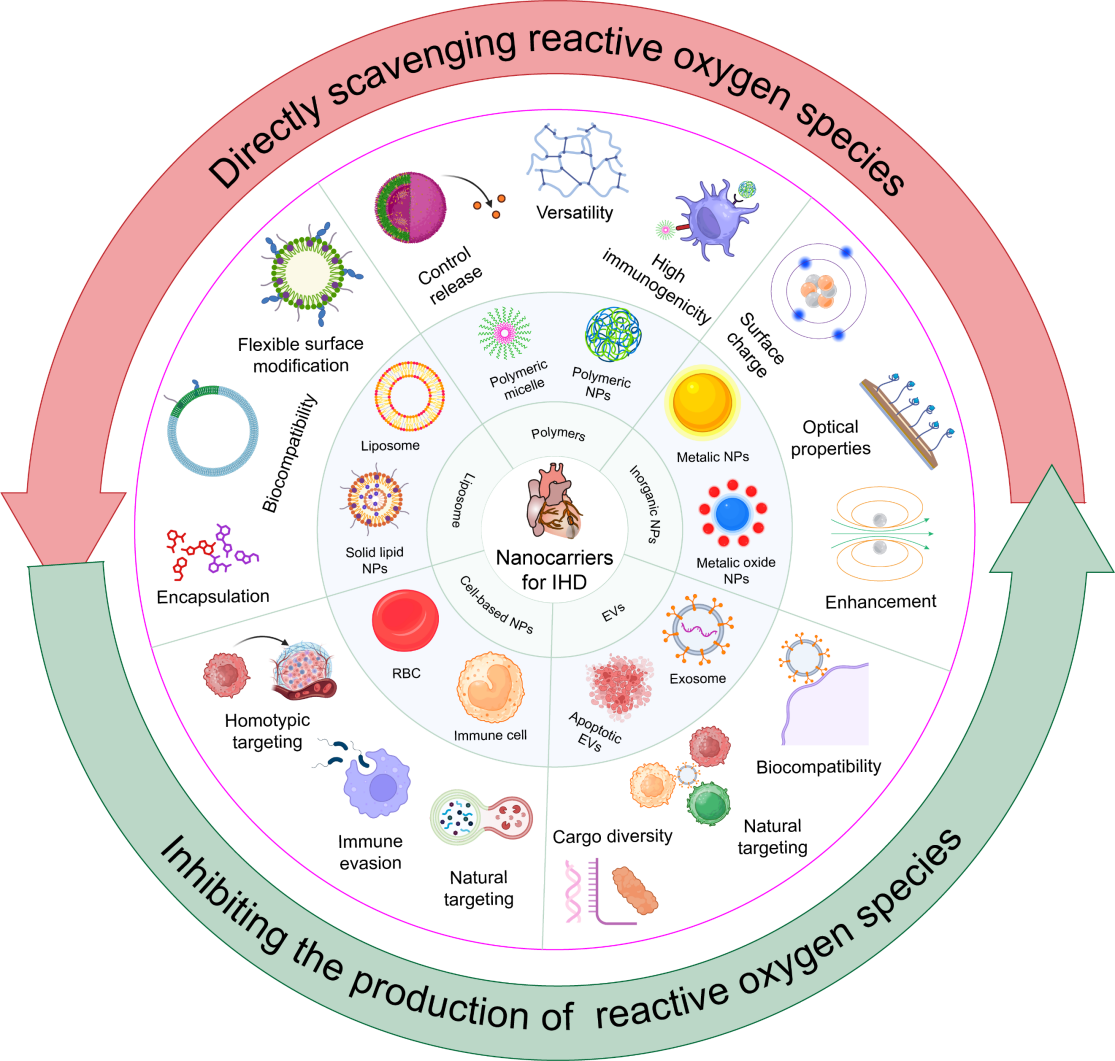
Illustration of the representative nanosystems used for IHD therapy. Different types of nanosystems offer distinct advantages, and selecting the most appropriate nanosystem depends on the specific needs of the therapy.

Nanomedicine, combining biomaterials and nanotechnologies, greatly improves traditional drug therapies for IHD 67, 68. By aligning with the unique pathogeneses and pathophysiological needs of various diseases, nanomedicine offers tailored therapeutic properties and functions. The customizable size, charge, and high surface-to-volume ratio of nanomedicines enable effective drug encapsulation, enhancing pharmacokinetics and pharmacodynamics in IHD treatment 69. Nanomedicines with multifunctional linkers, ligands, or coatings facilitate targeted delivery and controlled release, improving therapeutic outcomes in IHD 70. Due to their diverse chemical and physical properties, a wide variety of nanomaterials—including inorganic materials (e.g., gold NPs), organic materials (e.g., liposomes), biological molecules (e.g., proteins and peptides), and synthetic polymers—are utilized for nanocarrier fabrication, each offering unique advantages in drug delivery and therapeutic efficacy. This review presents advanced nanocarriers for treating IHD, such as liposomes, synthetic PNPs, inorganic NPs, EVs, and both cell-based and biomimicry-based nanocarriers (Figure 3). This highlights the key role of nanocarriers in improving the stability, targeting, and bioavailability of antioxidant drugs, thereby enhancing IHD treatment efficacy. It offers comprehensive references for future research in carrier selection.

### 4.1. Liposomes

Liposomes have emerged as a vital drug delivery system for IHD treatment due to their superior biocompatibility, ability to encapsulate both hydrophilic and hydrophobic drugs, controlled release properties, and enhanced molecular engineering capabilities 53. The inherent similarity between liposomes and cell membrane components provides them with excellent biocompatibility. This characteristic allows liposomes to be recognized and metabolized efficiently within the body 71.

Additionally, one of the key strengths of liposomes is their flexible surface modification potential. This capability enables the design of liposomes for targeted drug delivery and precise control over *in vivo* release. For example, Tal *et al.* successfully attached a ligand specific to the angiotensin II type 1 receptor (AT1) to liposomes, achieving accurate targeting of cardiac cells *in vitro* and after intravenous injection 72. This surface modification flexibility allows for the customization of liposomes to meet specific therapeutic requirements.

In addition to surface modification, liposomes are exceptional in their ability to encapsulate diverse therapeutic agents, including drugs, DNA, and diagnostic substances. This encapsulation not only protects the therapeutic cargo from degradation but also allows for controlled release in response to specific stimuli, such as abnormal pH levels and temperatures at pathological sites, thereby enhancing the targeted delivery and therapeutic efficacy 73. Polyethylene glycol (PEG)-coated liposomes further augment this benefit by offering superior stealth properties, extending drug circulation time in the bloodstream, and reducing adverse hemodynamic impacts 74.

The advantages of liposomes as versatile and efficient drug delivery vehicles. Their low toxicity and immunogenicity, coupled with their ability to be administered through multiple routes and forms, highlight their versatility in clinical applications 75. In summary, these ingenious molecular containers have become indispensable carriers in modern drug delivery.

### 4.2. PNPs

PNPs have garnered significant attention in nanomedicine due to their remarkable controlled release capabilities, versatility, and high immunogenicity 76. These attributes make PNPs highly effective for a range of therapeutic applications 77. Controlled release is a critical feature of PNPs, facilitated by biodegradable polymers such as poly (lactic acid) (PLA) and poly (lactic-co-glycolic acid) (PLGA). These polymers allow for precise timing and location of drug release, protecting encapsulated drugs from degradation and ensuring sustained therapeutic effects 78. This capability is particularly beneficial in treating IHD, where stable and prolonged drug activity is crucial.

The versatility of PNPs is another key advantage. They can be engineered to target specific tissues, cells, and subcellular structures, enabling precise and efficient drug delivery 79. This adaptability enhances the stability and activity of active components, ensuring that therapeutic agents are delivered at optimal concentrations. By precisely controlling surface characteristics and particle size, PNPs can regulate permeability, adjust solubility, and manage release patterns to meet specific therapeutic needs 80. High immunogenicity is a third significant benefit of PNPs. This property makes them excellent candidates for vaccine development and immunotherapy, as they can elicit robust immune responses 81. Overall, these properties position PNPs as crucial components in the development of innovative medical treatments. The ideal design of PNP delivery systems involves precise control of surface characteristics and particle size to regulate permeability, adjust solubility, enhance flexibility, and manage remedial release patterns, ensuring the desired therapeutic effects at the required times and locations.

### 4.3. Inorganic NPs

Inorganic NPs are highly effective nanoplatforms known for their unique surface charge, optical properties, and enhancement capabilities, making them suitable for a wide range of biomedical applications. Their composition, size, shape, and structure can be precisely tailored, utilizing their large surface area and distinct surface chemical properties to meet specific needs. In recent decades, there has been a significant increase in interest in these features for biomedical use 82.

One primary advantage of inorganic NPs is their surface charge, which is crucial for their interactions with biological molecules. Researchers can modify the surface coatings to enhance the colloidal stability of NPs in complex biological environments, ensuring effective dispersion in aqueous solutions. This is particularly important as the surface charge effects cellular uptake, distribution, and the overall biocompatibility of the NPs 83. Another key advantage is the optical properties of inorganic NPs. These NPs exhibit unique optical characteristics, such as plasmon resonance and fluorescence, which are invaluable for imaging and diagnostic applications. By adjusting the size and shape of the NPs, researchers can finely tune these properties, enabling high-resolution imaging and precise detection of biological targets 35.

Additionally, the enhancement potential of inorganic NPs is noteworthy. Surface engineering allows for precise control of interactions with biomolecules, leading to the development of efficient biomedical products. This capability is crucial for targeted drug delivery and improved therapeutic outcomes. However, the potential release of metal ions and the associated biological impact must be considered, as these could pose challenges for clinical applications due to potential adverse effects 84.

In summary, the surface charge, optical properties, and enhancement potential of inorganic NPs make them versatile and effective tools in biomedical research and applications. These attributes position inorganic NPs at the forefront of advancements in diagnosis, imaging, and targeted therapy, offering new possibilities for improving patient outcomes.

### 4.4. EVs

EVs, which include exosomes and microvesicles, are diverse membrane-bound entities originating from the endosomal system and plasma membrane. These vesicles, found in various biological fluids, play distinct roles in both physiological and pathological contexts, serving a dual role as therapeutic agents and delivery vehicles 85. One significant advantage of EVs is their biocompatibility. Because they originate endogenously, EVs are inherently compatible with human physiology, minimizing the risk of immunogenicity and adverse immune responses. This intrinsic compatibility makes EVs safer for clinical applications compared to many synthetic delivery systems 86.

Beyond their inherent components, EVs can transport a variety of small molecules, such as proteins and NAs, which can modulate the functions of recipient cells 86, 87. This versatile cargo-carrying capacity allows EVs to be employed in a broad range of therapeutic applications, from drug delivery to gene therapy. Another essential attribute of EVs is their natural targeting ability 85. They can efficiently traverse biological barriers, including tissue, cellular, and intracellular barriers, to reach and modulate specific target cells. Additionally, EVs can be engineered through genetic and chemical modifications to enhance their targeting specificity, thereby improving the precision of therapeutic interventions 88. Recent advancements have demonstrated the potential of genetically engineered hybrid nanovesicles (hNVs), which include cell-derived nanovesicles overexpressing high-affinity SIRPα variants, exosomes from human mesenchymal stem cells (MSCs), and platelet-derived nanovesicles. These hNVs significantly enhance macrophage phagocytosis of necrotic cells, mitigate inflammatory responses, and improve cardiac function in MI/RI models 89. However, the therapeutic application of exosomes also poses potential risks, including immunogenicity, tumor-promoting effects, limited understanding of long-term effects, and high associated costs. Additionally, when used as delivery vehicles, the challenge of removing endogenous cargos from EVs to avoid unwanted side effects remains significant 90.

### 4.5. Cell-based and biomimicry-based nanocarriers

Cell therapy is increasingly recognized as a promising strategy for treating ischemic diseases, with various cell types demonstrating efficacy in enhancing cardiac function post-ischemia 91. In addition to stem cell applications, the unique biological properties of various cell types are harnessed to develop sophisticated drug delivery strategies. Red blood cells (RBCs), neutrophils, monocytes, macrophages, and platelets are exemplary cell-based and biomimicry-based nanocarriers. These engineered carriers extend circulation times, target therapeutic sites, and overcome biological barriers, thereby enhancing drug delivery accuracy and intervention effectiveness 92. Recent advancements have shown the potential of human embryonic stem cell-derived epicardial cells (hEPs) in myocardial repair. These cells enhance CM survival, angiogenesis, and lymphangiogenesis by suppressing inflammatory responses mediated by type I interferon signaling 93. The advantages of cell-based and biomimicry-based nanocarriers stem from their capabilities in immune evasion, homotypic targeting, and functional integration.

Moreover, the cell membrane plays a crucial role in mediating interactions with the extracellular environment, such as signal transduction, recognition, adhesion, and immune modulation. Cell membrane coating is a promising technique that enhances the biointerfacial capabilities of nanocarriers, facilitating effective drug delivery to affected tissues 94. Given the intrinsic homing abilities of each cell type, cell membrane-coated NPs can be customized for targeted drug delivery, minimizing off-target effects and improving therapeutic efficiency. This method has been successfully applied to design biomimicry-based nanocarriers for targeted cardiovascular disease treatments, including IHD, atherosclerosis, and restenosis, demonstrating significant potential in cardiovascular therapies 95.

## 5. Nanotechnology in the diagnosis of IHD

Beyond their delivery roles, nanomedicines can also act as molecular probes for various imaging modalities, aiding in the localization and diagnosis of various disease processes. Advanced nanocarriers can be precisely engineered to improve the detection and imaging of ischemic tissues. These diagnostic nanomedicines can be functionalized with contrast agents for magnetic resonance imaging (MRI), computed tomography (CT), positron emission tomography (PET), and fluorescence imaging, ensuring accurate localization, characterization, and monitoring of ischemic lesions (Table 2).

### 5.1. MRI

MRI is a widely used noninvasive technique that provides high-resolution images of soft tissues. The incorporation of nanomedicines, especially those based on iron oxide, gadolinium (Gd), and manganese (Mn) as contrast agents, has significantly enhanced MRI capabilities. These cutting-edge *in vivo* imaging technologies enable real-time visualization of myocardial viability and the pathophysiology of myocardial ischemia.

Iron oxide NPs exhibit superparamagnetism, becoming magnetized only in the presence of an external magnetic field, making them ideal T2 contrast agents for MRI. The use of superparamagnetic iron oxide NPs (SPIONs) and ultrasmall superparamagnetic iron oxide NPs (USPIOs) has significantly enhanced the accuracy of MRI in diagnosing IHD. For example, Chen *et al.* reported that PP/PS@MIONs, superparamagnetic iron oxide NPs encapsulated with dual surfactants, improve detection of early-stage myocardial ischemia by targeting and accumulating in ischemic tissue, resolving inflammation, and enhancing MRI signals 36. Additionally, SPIONs have been extensively validated for their exceptional diagnostic performance in identifying IHD in clinical applications 96. These NPs significantly improve the precision of MI localization and detailed characterization, surpassing traditional imaging agents by providing clear, high-resolution images of infarcted tissue. Furthermore, SPIONs exhibit excellent biocompatibility and low toxicity, making them ideal for repeated clinical use. This innovation marks a significant breakthrough in the noninvasive diagnosis and monitoring of myocardial ischemia, promising improved patient outcomes through early and accurate detection.

Gd-based NPs, such as Gd-doped carbon dots (Gd-CDs), enhance MRI detection of IHD by providing dual magnetic resonance and fluorescence imaging. This technique overcomes the limitations of traditional Gd chelates, such as short circulation time, low relaxivity, and high dosage requirements. Gd-CDs offer precise imaging, renal clearance, and low cytotoxicity, making them promising for clinical application 97. Moreover, Mn ions are valuable for cardiac imaging due to their excellent paramagnetic properties and ability to enter CMs via L-type voltage-dependent Ca^2+^ channels, where they remain for several hours. Recently, Zheng *et al.* developed highly crystalline MnO NPs through the thermal decomposition of Mn oleate. These MnO-based NPs demonstrated high longitudinal relaxivity (*r*1) and relaxation rates without significant toxicity, making them promising for advanced cardiac imaging applications 98.

### 5.2. CT

CT is a pivotal imaging technique that provides detailed anatomical insights, particularly valuable in diagnosing IHD. CT scans offer rapid image acquisition, high-resolution details, and the ability to visualize both bone and soft tissues, making them essential in emergency settings for quick diagnosis and treatment planning 99. However, traditional CT imaging faces limitations such as exposure to ionizing radiation, potential allergic reactions to iodinated contrast agents, and challenges in differentiating soft tissue structures.

Nanomedicine holds the potential to significantly enhance the diagnostic capabilities of traditional CT imaging, particularly in the context of IHD. Gold NPs (AuNPs) are particularly promising due to their large scattering cross-section and low toxicity. They have been shown to improve imaging acquisition speed and reduce nephrotoxicity. For example, Kee *et al.* developed collagen-binding adhesion protein 35 (CNA35)-functionalized AuNPs for molecular imaging of myocardial scars. These CNA35-AuNPs demonstrated long blood circulation times and specific targeting capabilities to collagen in myocardial scars. In a rat MI/RI model, specific signal amplification in the myocardial scar was observed six hours after intravenous administration of CNA35-AuNPs, highlighting their potential for targeted imaging​. Despite these promising results, further studies on the biodistribution, toxicity, and biocompatibility of AuNPs in humans are necessary before they can be widely adopted in clinical practice 100.

### 5.3. PET

PET is a sophisticated imaging technique that employs radioactive tracers to produce detailed heart images, essential for diagnosing IHD. PET is known for its high sensitivity and specificity in detecting coronary artery disease (CAD) and assessing myocardial viability and perfusion. Nanomedicine offers significant enhancements to PET imaging for IHD diagnosis. Recent studies have highlighted the use of sodium [^18^F] fluoride (Na[^18^F]F) as a PET contrast agent to image MI/RI in rat models. Na[^18^F]F uptake was notably higher in infarcted areas, correlating with CM apoptosis and positive Ca^2+^ staining 101. These results demonstrate the potential of PET imaging for accurate MI/RI diagnosis​.

In addition, NP-based PET tracers can quantify myocardial blood flow (MBF) and myocardial flow reserve (MFR), providing valuable insights into the severity of ischemia and assisting in patient risk stratification 102. These advancements make PET imaging a powerful, noninvasive tool for diagnosing myocardial ischemia, essential for effective clinical decision-making. Future research should aim to optimize these NP tracers to enhance targeting, reduce toxicity, and improve imaging capabilities, ensuring their practical clinical application​.

### 5.4. Fluorescence imaging

MRI, CT, and PET are effective for tissue-level observation but have limitations in spatiotemporal resolution, preventing clear visualization of cellular changes in the pathophysiological microenvironment. In contrast, fluorescence imaging offers high spatiotemporal resolution and sensitivity, making it superior for noninvasive and precise monitoring of IHD.

Fluorescently labeled NPs, such as fluorescence-labeled CD47-EVs (CD47-EVs), are used to track drug distribution and delivery in myocardial tissues. These CD47-EVs have shown prolonged circulation times and preferential accumulation in the myocardium, providing real-time monitoring of therapeutic effects and drug delivery efficiency 103. Similarly, Yang *et al.* developed a fluorescent SiO_2_@PDA-DNA-CeO_2_ nanocomposite for detecting exogenous molecules in MI/RI models. This nanocomposite allowed simultaneous detection of intracellular miRNA and H_2_O_2_* in vivo*, specifically targeting apoptotic CMs, and revealing significant miR-21 expression in response to oxidative stress 104.

Further advancements include Gd ferrate and trigadolinium pentairon (III) oxide NPs (GF/TPO NPs) grafted with fluorescent pigment indocyanine green (ICG). These NPs efficiently accumulated in infarcted areas, enhancing vascular permeability and providing clear fluorescence signals in ischemic tissues. This dual imaging capability facilitates the detailed visualization of IHD and assessment of therapeutic interventions 105.

These imaging methods collectively enhance the precision, sensitivity, and specificity of IHD diagnosis, each with unique benefits tailored to different diagnostic needs. Future research should focus on optimizing these NP-based imaging agents for better targeting, reduced toxicity, and enhanced imaging capabilities. This will ensure their effective clinical application and improve patient outcomes through precise and early diagnosis of ischemic heart conditions.

## 6. Antioxidant nanomedicines for IHD treatment

ROS are crucial targets in IHD, as they perpetuate a vicious cycle of inflammation and Ca^2+^ overload 6. Antioxidant therapy, which utilizes antioxidants to neutralize excessive ROS, not only mitigates oxidative stress but also limits subsequent cell apoptosis and inflammatory responses, making it a preferred strategy for treating IHD. Preclinical studies have explored a range of antioxidants, including antioxidant enzymes, nonenzymatic antioxidants, NSMs, inorganic and other substances 106. However, their poor solubility, short half-life, and limited bioavailability significantly impede their potential for clinical translation 107, 108. To address these challenges, multifunctional nanocarriers can improve this strategy by enhancing the pharmacokinetic properties of antioxidants and facilitating their accumulation in damaged cardiac tissue. This approach offers a promising perspective for the clinical treatment of IHD. In this section, we highlight the recent progress in nanomedicines targeting oxidative regulation, categorizing and summarizing key studies based on their antioxidant mechanisms (Table 3).

### 6.1. Antioxidant enzyme-based nanomedicines

Oxidative stress, while essential for cellular signaling and homeostasis at low concentrations, becomes harmful at elevated levels 25. Myocardial ischemia impairs oxidative phosphorylation, leading to a dysregulated oxidative environment within cells 109. Intracellular antioxidant enzymes are the primary defense against oxidative imbalance, but they are often overwhelmed by severe oxidative challenges 110. Therapeutically enhancing these enzymes could bolster cellular resilience and mitigate oxidative injury.

**Figure 4:**
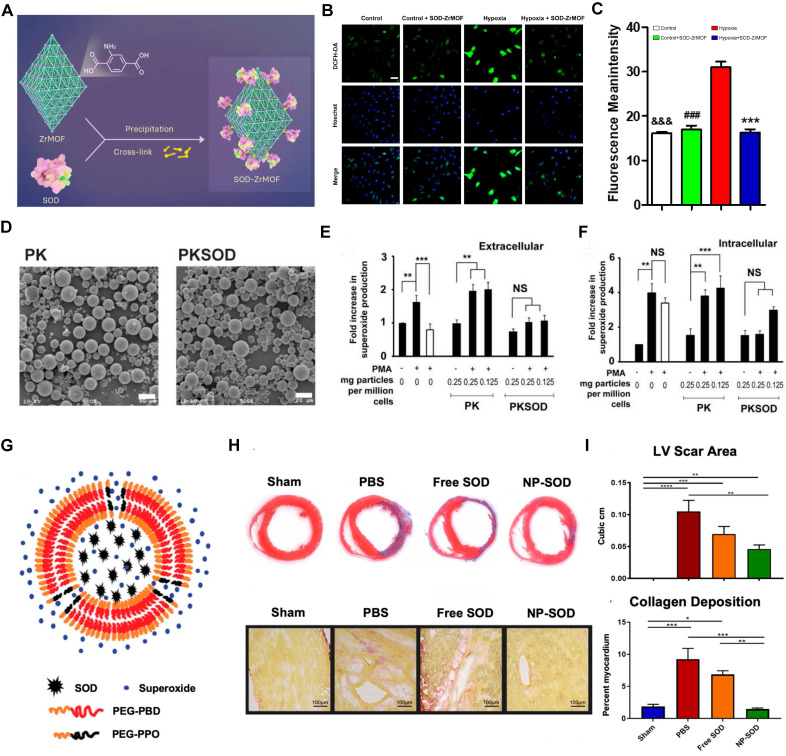
(A) Schematic of the construction of SOD-ZrMOF. (B-C) The ROS level in CMs. Adapted with permission from 112, copyright 2022 Elsevier. (D) Representative SEM of empty polymer (PK) and PKSOD. (E-F) Extracellular and intracellular superoxide concentration. Adapted with permission from 107, copyright 2010 Elsevier. (G) Schematic of PEG-PBD polymer. (H-I) Masson's Trichrome and Picrosirius red staining with quantitative analysis for the respective images. Adapted with permission from 113, copyright 2021 John Wiley and Sons.

Metal-organic frameworks (MOFs) are crystalline materials composed of metal ions or clusters coordinated to organic ligands, forming a porous structure. Due to their highly tunable porosity, large surface area, and versatile functionality, MOFs have garnered significant interest in various applications, including gas storage, catalysis, and drug delivery 111. Guo *et al.* developed the SOD-ZrMOF nanoconstruct, integrating SOD with a zirconium-based framework, which exhibits exceptional biocompatibility and effectively neutralizes ROS (Figure 4A). These NPs present advanced approaches for MI repair, promising enhanced cardiac recovery post-ischemia after intracardiac administration 112. Concurrently, SOD encapsulated within microparticles has shown protective effects against MI/RI in rat hearts, reducing superoxide-mediated damage (Figure 4B-C). Although Zr-MOFs exhibit superior efficacy compared to that of native SOD proteins, they face challenges such as stability issues, complex synthesis, potential toxicity, and limited functionalization. The application of MOFs in antioxidant enzyme delivery requires further investigation into their long-term biocompatibility and degradation within the body. Research should also explore the potential of integrating MOFs with other nanomaterials to form hybrid systems that can provide more controlled release profiles and target multiple oxidative stress pathways simultaneously. Additionally, the role of these nanomedicines in complex *in vivo* environments, including interactions with immune cells and other components of the ischemic tissue, needs to be better understood to optimize therapeutic outcomes. In contrast, polymer-based nanocarriers offer superior biocompatibility, customizable drug release profiles, enhanced stability, versatile functionalization, and ease of large-scale manufacturing 76, 77. For instance, researchers have encapsulated SOD1 within the economically feasible and more stable poly (cyclohexane-1,4-diyl acetone dimethylene ketal) (PCADK) polymer (Figure 4D), resulting in PKSOD, which reduces the concentration of superoxide both intracellularly and extracellularly (Figure 4E-F) 107.

The dense structure of polymeric carriers makes it challenging for ROS to directly interact with the encapsulated drugs, hindering rapid onset of antioxidative effects. To address this, Atluri and colleagues incorporated the diblock copolymer PEG-PPO into the PEG-PBD polymer, resulting in a nanocarrier that facilitates the delivery of unmodified enzymes, protects against proteolysis, and allows access to ROS via a highly porous membrane (Figure 4G). This construction accommodates and retains antioxidant enzymes within the NPs while allowing small molecules, such as free •O_2_^-^, to pass through the polymer's permeable membrane into the carrier interior. Research indicates that the SOD-encapsulated PEG-PBD polymer can reduce the area of MI and diminish negative cardiac remodeling (Figure 4H-I) 113. These results suggest that exogenous supplementation of antioxidant enzymes can facilitate myocardial repair by modulating oxidative balance. However, while SOD quenches •O_2_^-^ to combat oxidative stress, the resulting H_2_O_2_ presents new challenges for clinical application, necessitating the concurrent use of CAT to detoxify H_2_O_2_.

### 6.2. Nonenzymatic-based nanomedicines

Nonenzymatic antioxidants typically react rapidly within the body to neutralize various types of free radicals, thereby mitigating the damage caused by oxidative stress. Dopamine, a crucial neurotransmitter present in the brain, plays a key role in regulating emotions, reward mechanisms, and pleasure 114. Research has shown that dopamine can be polymerized into PDA through a process called oxidative polymerization under alkaline conditions, involving oxidation and cyclization steps that lead to the formation of melanin-like structures 115. In addition, PDA has been identified as an antioxidant that can inhibit inflammation and oxidative stress 116.

**Figure 5:**
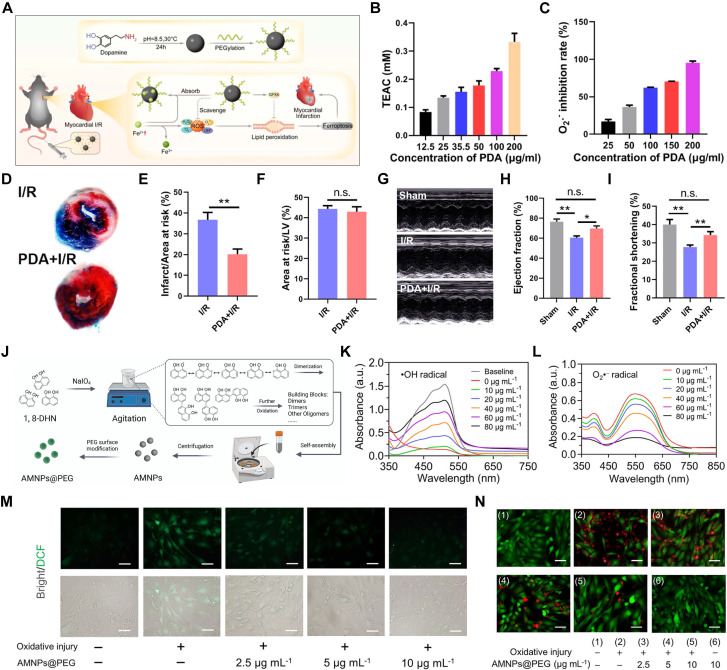
(A) Schematic diagram of the ROS-scavenging and Fe^2+^-chelating abilities of PDA NPs. (B) ABTS measurement of the antioxidant capacity of PDA NPs. (C) •O_2_^-^-scavenging activity of PDA NPs. (D) Representative Evans Blue and TTC stained heart tissue sections. (E-F) The infarct size relative to area at risk (AAR) and the AAR relative to the area of left ventricle (LV). (G) Representative M-mode echocardiograms of myocardial I/R mice exposed to different treatments collected 24 h postoperatively. (H-I) Calculations of ejection fraction and fractional shortening percentages. Adapted with permission from 106, copyright 2021 American Chemical Society. (J) Schematic illustration of the fabrication procedure of AMNPs. (K-L) Representative UV-vis absorption results of AMNPs on scavenging •OH and •O_2_^-^. (M) Representative fluorescent images of intracellular ROS levels. (N) Fluorescence images of myocardial cell activity. Adapted with permission from 120, copyright 2022 Elsevier.

Li's team employed PEG-modified PDA NPs as cardioprotective agents to alleviate MI/RI in mice (Figure 5A). Unlike typical nanocarriers, PDA NPs themselves exhibit inherent antioxidant properties, effectively scavenging ROS such as •OH and •O_2_^-^ (Figure 5B-C). Intravenous injection of PDA NPs into mice with MI/RI reduced infarct size (Figure 5D-F) and improved cardiac function (Figure 5G-I) 106. Although PEGylation can extend the blood circulation time of PDA NPs to some extent; however, cell membrane coatings are superior to PEGylation in prolonging blood circulation time due to their enhanced biocompatibility, natural immune evasion properties, and improved functionalization potential. The biomimetic nanoplatform (PDA@M) created by coating PDA NPs with macrophage membranes not only reduces the clearance of PDA NPs by monocytes/macrophages but also enhances their targeting ability to CMs, thereby providing more precise antioxidant therapy 117.

The design of PDA is fundamentally based on emulating the properties of natural melanin. Unlike melanin, which is challenging to extract and purify, PDA provides enhanced stability, scalability, and multifunctionality, making it ideal for various biomedical applications 118. Additionally, researchers are actively pursuing breakthroughs in new natural melanin analogs. Allomelanins, a novel melanin analog synthesized by fungi using non-nitrogenous and non-sulfurous 1,8-dihydroxynaphthalene (1,8-DHN), has shown considerable potential. Research indicates that 1,8-DHN can form nanomedicines with robust HAT properties, providing potent antioxidant effects 119. PEG-modified allomelanin NPs (AMNPs@PEG) have been developed for targeted therapy of MI/RI (Figure 5J). AMNPs@PEG demonstrated significant efficacy in scavenging •OH and •O_2_^-^ *in vitro* (Figure 5K-L). Further cellular studies confirmed the protective role of AMNPs@PEG in CMs, as AMNPs@PEG effectively alleviated intracellular ROS and ROS-induced damage (Figure 5M-N) 120.

Unlike PDA, which primarily quenches ROS through electron transfer redox mechanisms, bilirubin functions by scavenging peroxyl radicals and directly neutralizing ROS. PEGylated bilirubin facilitates the self-assembly of bilirubin NPs (BRNPs), which effectively target the MI/RI site. Research indicates that BRNPs mitigate oxidative stress and inflammation, suggesting a novel therapeutic avenue for MI/RI 24.

While PDA and bilirubin can naturally self-assemble into NPs, many nonenzymatic antioxidants require appropriate carriers for effective delivery. Among various exogenous carriers, EVs have recently emerged as a popular candidate. One of the key advantages of EVs over artificial delivery systems is their inherent biocompatibility and natural origin. Researchers engineered EVs derived from adipose-derived stem cells (ADSCs) to load Mel, creating Mel@NVs. The impact of Mel@NVs on cellular oxidative stress and MI repair was studied. The results indicated that treatment with Mel@NVs under ischemic conditions reduced cell apoptosis from 42.59 ± 2.69% to 13.88 ± 1.77%. Furthermore, Mel@NVs ameliorated excessive ROS generation, promoted microvessel formation, and attenuated cardiac fibrosis 121.

CoQ10 is a vital endogenous antioxidant that plays a crucial role in oxidative phosphorylation 122. Previous research has demonstrated the potential of CoQ10 in treating and preventing IHD, hypertension, hyperlipidemia, CAD, and HF 123, 124. However, due to the high lipophilicity and low solubility of CoQ10, delivering it within cells presents challenges. Liposomes, as nanoscale drug delivery carriers, have been proven to enhance the therapeutic efficacy of CoQ10. A research team in the United States has employed various methods to prepare CoQ10-loaded liposomes, optimizing the formulation to achieve maximum payload and stability. These CoQ10-containing liposomes have shown significant cardioprotective effects against MI/RI, offering a new approach to protecting the ischemic myocardium 53.

Alpha-lipoic acid (LA) is a natural antioxidant compound featuring two thiol groups that can be oxidized or reduced, allowing it to participate in various redox biochemical reactions 125. However, its therapeutic application is limited by rapid and extensive distribution, high metabolic clearance, a short half-life of approximately 25 minutes, and a high systemic pre-clearance rate 126. Incorporating LA into a PLGA copolymer and forming a film via electrospinning technology (referred to as LA@PLGA) significantly improves these limitations. LA@PLGA enables controlled release, enhancing the stability and bioavailability of LA. Studies have demonstrated that LA@PLGA exhibits potent antioxidant and anti-apoptotic effects in primary CMs treated with H_2_O_2_, and its application to the surface of the heart in mice with MI significantly improves cardiac function and reduces cardiac fibrosis throughout the ventricular remodeling process 127.

In summary, nanocarriers play a crucial role in enhancing antioxidant therapy for IHD by improving the stability, bioavailability, and targeted delivery of nonenzymatic drugs, thus addressing the limitations of traditional therapies. These advanced carriers, including liposomes and polymeric systems, exhibit excellent biocompatibility and customizable drug release profiles, enabling precise therapeutic effects. Additionally, some nonenzymatic antioxidant drugs can self-assemble into nanostructures, and with simple modifications, their properties can be further enhanced to achieve better therapeutic outcomes. Future research should focus on overcoming the production and therapeutic challenges of nonenzymatic nanomedicines, enhancing the targeting, scalability, and consistency of nanomedicines, and conducting extensive *in vivo* studies to understand their long-term effects. The advancement of nonenzymatic antioxidant nanomedicines could benefit from exploring synergies with other therapeutic agents, such as anti-inflammatory molecules or growth factors, to enhance the overall therapeutic effect. The potential of these nanomedicines in long-term treatment and chronic disease management should also be investigated, focusing on their impact on tissue remodeling and regeneration post-injury. Furthermore, the development of next-generation biomimetic nanoplatforms that closely mimic the native cellular environment could offer more effective and targeted therapies.

### 6.3. NSM-based nanomedicines

Small molecular compounds, derived from both natural sources and synthetic modifications, are integral to the prevention and treatment of various diseases due to their potent antioxidant effects 76. Small molecular antioxidants like curcumin, resveratrol (RSV), baicalin (BN), and quercetin (Que) are effective in combating oxidative stress but face significant delivery challenges, such as poor solubility, rapid metabolism, and lack of specific targeting. Nanotechnology-based delivery systems have revolutionized their clinical potential, ensuring more effective therapeutic outcomes.

Que, a typical flavonoid belonging to the flavonol class, has been confirmed to provide robust cardioprotection in IHD by modulating inflammation, oxidative stress, and CM apoptosis 128. However, its clinical application is limited due to its low water solubility, short half-life, poor transference, and low bioavailability. Mesoporous silica NPs (MSNs) have emerged as a promising platform for drug delivery due to their unique structural and functional properties. One of the primary advantages of MSNs is their high surface area and large pore volume, allowing for substantial drug loading capacity. The tunable pore size and surface chemistry of MSNs enable precise control over drug release kinetics, enhancing the therapeutic efficacy of encapsulated drugs. Encapsulating que within MSNs has been shown to extend its half-life *in vivo*, enhancing drug absorption. Both *in vitro* and *in vivo*, que-loaded MSNs (Q-MSNs) exhibit superior efficacy in inhibiting apoptosis and oxidative stress, reducing MI size, and improving ventricular remodeling and cardiac function, compared to que alone 62.

**Figure 6:**
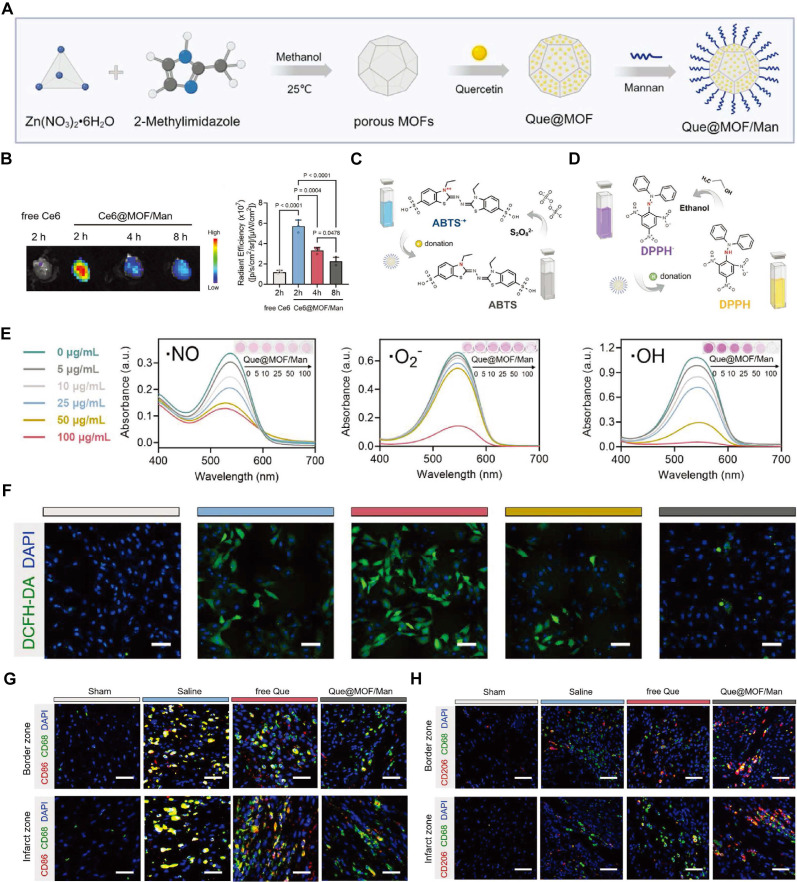
(A) Schematic diagram of the construction of Que@MOF/Man. (B) Representative ex vivo fluorescence images and quantification analysis. (C-D) Figure illustrating the scavenging efficacy of Que@MOF/Man against ABTS^•+^ (A) and DPPH^•^ radicals. (E) UV-Vis spectra depicting the radical scavenging activities of Que@MOF/Man against •NO, •O_2_^-^, and •OH radicals. (F) Representative images of ROS production. (G) Representative images of CD68+ macrophages. (H) Representative images of CD206+ macrophages. Adapted with permission from 38, copyright 2021 John Wiley and Sons.

In comparison to metal nanocarriers, MOF nanocarriers exhibit significant advantages, such as tunable porosity, biocompatibility, and stimuli-responsive release, making them highly effective for antioxidant delivery 111. For instance, the encapsulation of Que within these multifunctional nanocarriers has demonstrated improved stability and bioavailability (Figure 6A). Que@MOF/Man facilitated real-time imaging of the therapeutic process, providing critical insights into drug distribution and efficacy, thereby paving the way for personalized medicine in treating MI/RI (Figure 6B). Que@MOF/Man exhibited a concentration-dependent scavenging effect on •NO, •O_2_^-^, and •OH radicals, indicating the effective elimination of these harmful free radicals by Que@MOF/Man (Figure 6C-E). Interestingly, co-culturing with Que@MOF/Man-treated macrophages appears to inhibit ROS production in injured cardiomyocytes (Figure 6F). Additionally, *in* vivo studies have shown that Que@MOF/Man significantly mitigate inflammation (Figure 6G-H), leading to a marked reduction in infarct size 38.

However, despite these advantages, the biodegradation and excretion of metal-based materials need to be thoroughly understood to ensure long-term safety. Therefore, using nanocarriers with good biodegradability *in vivo* may be a more favored choice. For instance, Lu *et al.* developed a novel RGD modified and PEGylated nanostructured lipid carrier (RGD/PEG-PUE-SLN) for puerarin (PUE), which enhances its bioavailability *in vivo*, and amplifies its protective effects. *In vitro* studies assessing the cytotoxicity of PUE-loaded SLN and free PUE at varying concentrations revealed that the RGD/PEG-PUE-SLN, PEG-PUE-SLN, and PUE-SLN groups showed no significant cytotoxicity relative to the saline control, underscoring the superior biocompatibility of SLN. In *in vivo* applications, it was evident that the RGD/PEG-PUE-SLN formulation resulted in the smallest infarct size among all the formulations tested 129. Researchers have taken advantage of the properties of liposomes to design nanocarriers for various small-molecule drugs. For example, Han's team encapsulated poorly water-soluble Schisandrin B (Sch B) into PEG-modified solid lipid NPs (SLNs). They also modified the liposome surfaces with matrix metalloproteinase (MMP)-targeting peptides, creating MMP-Sch B SLNs nanomedicines. The profile demonstrated that the plasma concentration of Sch B solution decreased rapidly and was cleared from circulation within 4 hours. In contrast, Sch B loaded SLNs exhibited a significantly prolonged plasma circulation time, indicating that liposomes can effectively extend the circulation time of Sch B. The prolonged blood circulation time facilitates drug accumulation in the heart, thereby reducing the required dosage and improving therapeutic outcomes 73. Furthermore, researchers developed novel panax notoginsenoside (PNS)-loaded core-shell hybrid liposomal vesicles (PNS-HLV) to overcome the limited bioavailability of PNS and enhance its protective effects. PNS-HLV demonstrated controlled drug release profiles, suggesting promising prospects for improving the bioactivity of free drugs 130.

**Figure 7:**
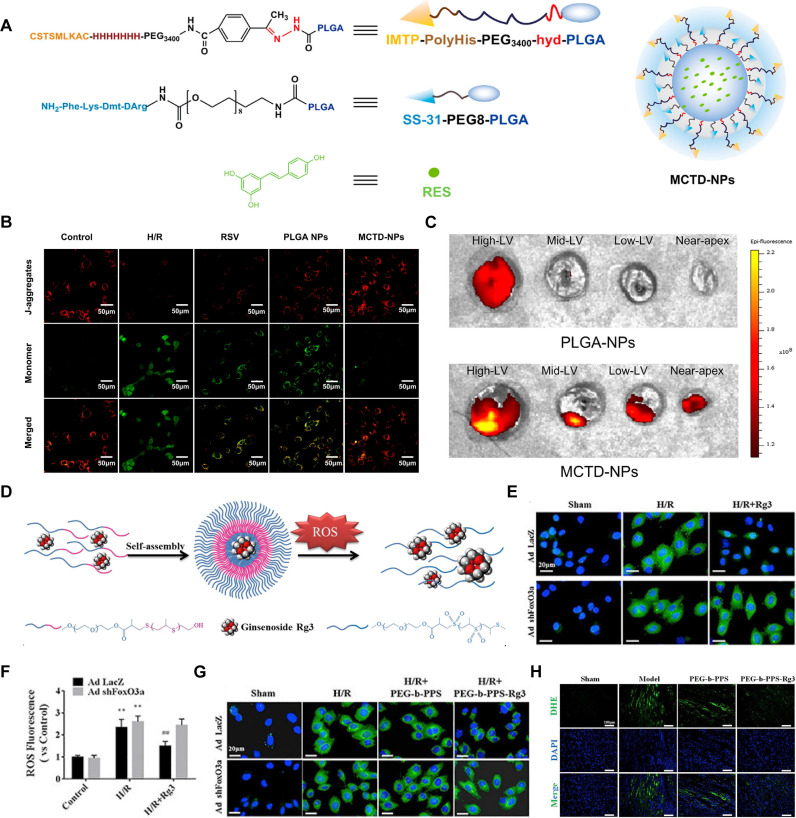
(A) Schematic diagram of the construction of MCTD-NPs. (B) Representative image of mitochondrial membrane potential. (C) Ex vivo distribution of cy7.5 labeled MCTD-NPs. (E) Evans Blue and TTC staining. Adapted with permission from 78, copyright 2019 Elsevier. (D) Schematic diagram of the construction of PEG-b-PPS-Rg3. (E-F) Representative ROS staining and quantification after administration Rg3. (G) Representative ROS staining with or without knockdown of FoxO3a after administration PEG-b-PPS-Rg3. (H) Representative DHE staining. Adapted with permission from 132, copyright 2019 Elsevier.

In addition to liposomes, PLGA is a nanocarrier material known for its excellent biodegradability and high safety in applications. Zhou *et al.* designed a multistage targeted drug delivery system, named MCTD-NPs, by encapsulating RSV in PLGA NPs modified with ischemic myocardial-targeting peptide (IMTP) and SS-31 (Figure 7A). *In vitro* cell experiments indicated that MCTD-NPs were more readily absorbed by H/R-injured H9c2 cells compared to PLGA-NPs and RSV. The findings revealed that MCTD-NPs were more effective in delivering RSV to H/R injured H9c2 cells compared to PLGA-NPs, which were less effective. The SS-31 modification enabled further mitochondria accumulate of RSV, restoring membrane potential and reducing ROS production caused by mitochondrial dysfunction (Figure 7B). *In vivo* cardiac targeting studies revealed that MCTD-NPs accumulate significantly in the hearts of MI/RI rats compared to PLGA-NPs (Figure 7C). The increased cardiac specificity of the MCTD-NPs significantly reduced the percentage of infarct size, apoptosis, and inflammation in the ischemic myocardium, and stabilized mitochondrial function 78.

Compared to synthetic nanodelivery systems, EVs provide superior biocompatibility and safety. EVs are naturally secreted by cells, making them highly compatible with the human body and less likely to induce an immune response or toxicity 86. Curcumin loaded into DOPE-PEG-CTP-modified MSC-derived exosomes can greatly improve its heart-targeting ability and antioxidant therapeutic outcomes 131. This targeting capability is especially beneficial for the precise delivery of antioxidants to ischemic myocardial tissues, thereby reducing off-target effects and enhancing therapeutic outcomes.

Choosing biocompatible nanocarrier materials is essential, but addressing drug toxicity due to off-target effects is equally crucial. This can be effectively managed through the rational design of nanocarriers. ROS-responsive nanocarriers provide a sophisticated solution by significantly reducing drug toxicity. These carriers are designed with linkers or materials that degrade in the presence of ROS, ensuring that drug release is confined to pathological environments where ROS levels are elevated. This targeted release mechanism minimizes systemic drug distribution, thereby reducing off-target effects and associated toxicity. By specifically responding to the oxidative stress in damaged tissues, ROS-responsive nanocarriers enhance therapeutic efficacy while mitigating adverse effects. Thus, an amphiphilic block copolymer composed of hydrophilic PEG and hydrophobic poly (propylene sulfide) (PPS) was fabricated as an excellent carrier for Ginsenoside Rg3 (PEG-b-PPS-Rg3) (Figure 7D). Under ROS conditions, PPS transitions from hydrophobic to hydrophilic upon oxidation, causing the polymer to disassemble and release the encapsulated Rg3. ROS fluorescence staining indicated that Rg3 significantly reduced ROS levels induced by H/R in H9C2 cells (Figure 7E-F). The antioxidative effect of Rg3 was abolished after FoxO3a was knocked out, indicating that Rg3 exerts its effects by modulating FoxO3a (Figure 7G). Upon intracardiac injection, PEG-b-PPS-Rg3 significantly inhibited the generation of ROS within the injured myocardium, thereby reducing infarct size (Figure 7H) 132.

In summary, nanomedicines derived from NSMs show great potential in treating IHD by effectively reducing oxidative stress. Despite the significant therapeutic effects observed in these nanoformulations, there is a need for further exploration of their long-term safety and biocompatibility. Current challenges include optimizing drug formulations to suit different stages of IHD and diverse patient profiles, as well as refining nanocarrier designs to enhance delivery efficiency and minimize adverse reactions. Additionally, integrating real-time monitoring capabilities within these nanocarriers could provide clinicians with valuable feedback on drug efficacy and tissue response, enabling more personalized treatment regimens. Future research should also consider the scalability of these nanomedicines and their compatibility with current clinical practices to facilitate smoother transitions from bench to bedside.

### 6.4. Inorganic nanomedicines

Recently, inorganic nanomedicines have garnered considerable interest due to the intriguing electronic properties of transition metals 133. These nanomedicines exhibit redox, optical, and magnetic characteristics, making them highly effective agents for treating IHD 134. Constructed from inorganic metals, these nanomedicines function primarily by emulating the activity of antioxidant enzymes and are termed "nanozymes". Compared to natural enzymes and other nanomaterials, nanozymes offer superior catalytic activity, selectivity, stability, and a wider substrate range, addressing many of the limitations associated with natural enzymes 135. Nanozymes demonstrate a robust capacity to neutralize various free radicals, with some even displaying multi-enzyme activities. This enables them to efficiently eliminate ROS through cascade reactions with minimal use 136. The inherent nanostructure and catalytic capabilities of nanozymes facilitate direct drug application. Furthermore, the activity of nanozymes can be modulated by targeting molecules, facilitating both passive and active targeting of injured myocardial sites. This targeted approach effectively protects ischemic myocardial tissue and reduces infarct size.

**Figure 8:**
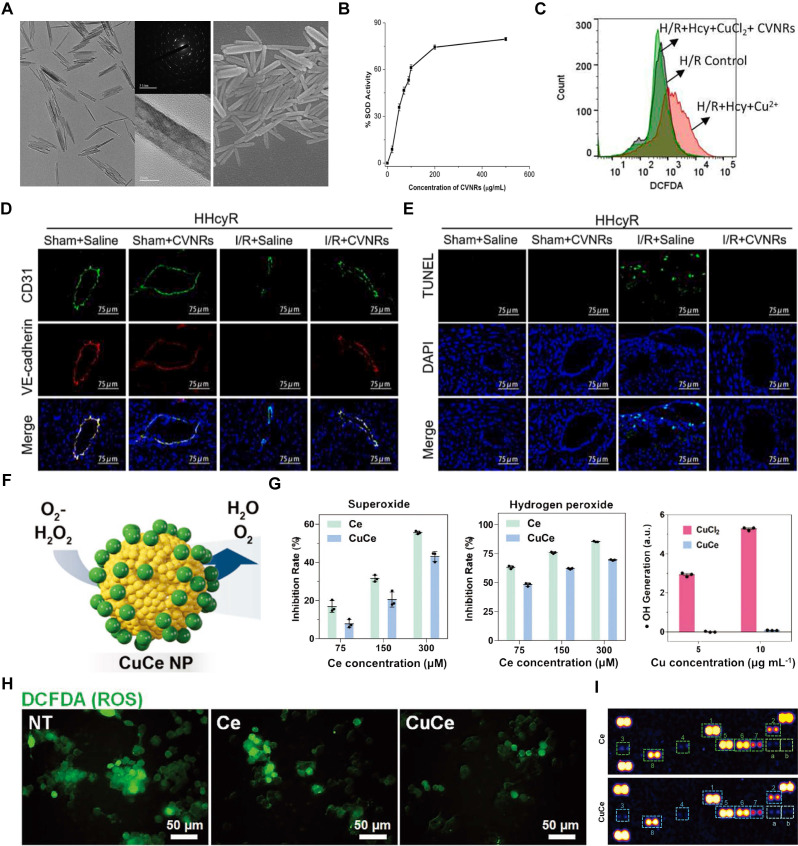
(A) TEM images of CVNRs. (B) The SOD-like activity of CVNRs. (C) Flow cytometric analysis of ROS levels in HCMECs. (D) Immunofluorescence of VE-cadherin. (E) TUNEL assay. Adapted with permission from 138, copyright 2024 Elsevier. (F) Schematic illustration of the function of CuCe NPs. (G) ROS scavenging effect of Ce NPs and CuCe NPs. (H) Representative fluorescence images of ROS staining. (I) Expression and quantification of immunomodulatory cytokines analyzed with mouse cytokine array. Adapted with permission from 139, copyright 2023 John Wiley and Sons.

Cerium (Ce)-containing nanozymes have attracted widespread attention due to their ability to undergo a valence transition between the Ce^3+^ (reduced) and Ce^4+^ (oxidized) forms, exhibiting significant SOD-like activity 137. Zhang's team reported a type of Ce vanadate nanorods (CVNRs) that possess superior SOD-like activity and act as effective ROS scavengers (Figure 8A). The SOD-like activity of CVNRs is attributed to the redox-active Ce centers and structure-stabilizing V centers (Figure 8B). This SOD-like activity effectively reduces intracellular ROS levels in human cardiac microvascular endothelial cells (HCMECs), protecting against Hcy and Cu^2+^-induced cell death under H/R conditions (Figure 8C). *In vivo* experiments showed that a 20 mg/kg CVNRs treatment significantly restored cardiac microvascular integrity and decreased apoptosis in microvascular endothelial cells (Figure 8D-E). These findings suggest that CVNRs are potent ROS scavengers for treating MI/RI 138.

While SOD converts •OH and •O_2_^-^ into H_2_O_2_, the resulting H_2_O_2_ can still cause cellular damage. To address this, researchers combined SOD with CAT. They synthesized copper-deposited Ce NPs (CuCe NPs), where Ce NPs serve as antioxidant carriers, transporting and delivering copper ions through biotransformation, enabling dual enzymatic catalysis similar to SOD1 and CAT (Figure 8D). In antioxidant assays, CuCe NPs displayed slightly lower performance than Ce NPs with the same Ce content, likely due to copper deposition masking the Ce surface (Figure 8E). However, CuCe NPs demonstrated a greater ability to scavenge ROS compared to Ce NPs (Figure 8F). The removal of ROS supports the transition of macrophages to an anti-inflammatory phenotype. Dot blot analysis indicated that CuCe NPs significantly reduced the expression of most proinflammatory cytokines compared to Ce NPs. The expression levels of representative pro-inflammatory cytokines (TNF-α and iNOS) and an anti-inflammatory cytokine (TGF-β) showed that CuCe NP treatments mitigated these effects compared to the non-treated group (Figure 8G) 139. The antioxidant and anti-inflammatory effects of CuCe NPs significantly enhance their therapeutic efficacy in treating IHD.

**Figure 9:**
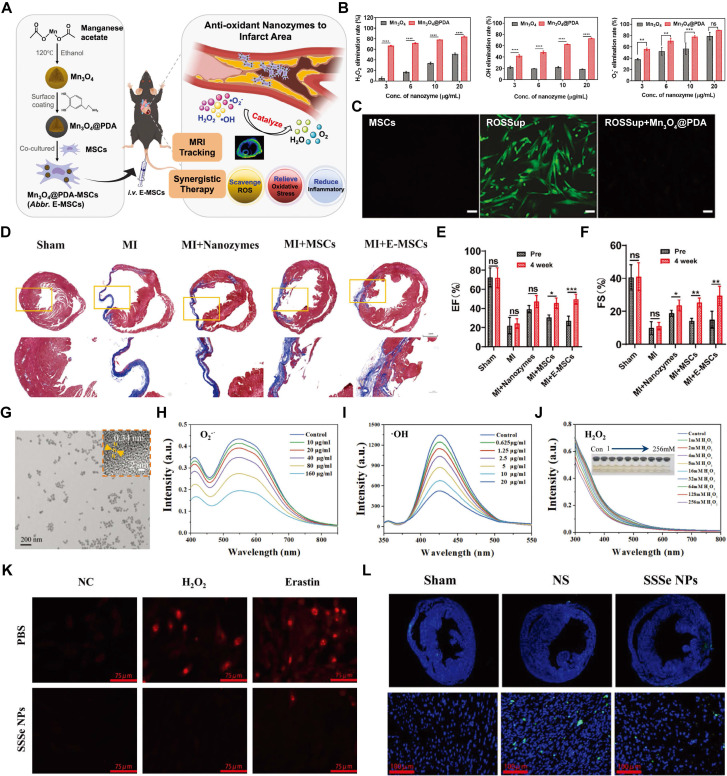
(A) Diagram showing the preparation of Mn_3_O_4_@PDA-MSCs and their use in *in vivo* MRI tracking and enhancement of the microenvironment for improved MI therapy. (B) ROS scavenging effect of Mn_3_O_4_ and Mn_3_O_4_@PDA-MSCs. (C) Representative fluorescent images of ROS levels in MSCs. (D) Representative images of Masson's trichrome staining. (E-F) Ejection fraction (EF%) and fractional shortening (FS%). Adapted with permission from 140, copyright 2024 John Wiley and Sons. (G) TEM image of SSSe NPs. (H-J) ROS scavenging effect of SSSe NPs. (K) MitoSOX staining. (L) Representative fluorescence images of TUNEL staining. Adapted with permission from 141, copyright 2022 John Wiley and Sons.

In addition to Ce-based nanozymes, Mn can also be utilized to construct SOD-mimicking nanozymes, such as Mn_3_O_4_ nanozyme. These nanozymes mimic the natural antioxidant functions of SOD by efficiently catalyzing the dismutation of •O_2_^-^ into O_2_ and H_2_O_2_, thereby reducing oxidative stress in IHD. The catalytic properties of Mn_3_O_4_ nanozymes are derived from their unique crystal structure and oxidation states, which facilitate electron transfer processes essential for ROS neutralization. Liu *et al.* modified the surface of Mn_3_O_4_ nanozyme with PDA through a polymerization reaction, creating Mn_3_O_4_@PDA (Figure 9A). This modification increased the biocompatibility of Mn_3_O_4_, improving its interaction with stem cells and enhancing its SOD-mimicking activity. The scavenging capacities of Mn_3_O_4_ and Mn_3_O_4_@PDA for H_2_O_2_, •OH, and •O_2_^-^ were evaluated. Compared to Mn_3_O_4_, Mn_3_O_4_@PDA showed a significantly higher rate of ROS elimination (Figure 9B). The antioxidant effect of Mn_3_O_4_@PDA significantly reduced the ROS concentration in MSCs (Figure 9C). In addition, *in vivo* studies have demonstrated that Mn_3_O_4_@PDA nanozymes possess reliable cardiac protective effects, evidenced by reduced infarct size and improved recovery of cardiac function (Figure 9D-F) 140.

In a recent study, researchers developed an innovative self-sustaining antioxidant strategy to treat MI using self-sustaining selenium-embedded NPs (SSSe NPs) (Figure 9G). These SSSe NPs exhibited potent scavenging effects on •O_2_^-^, •OH, and H_2_O_2_ (Figure 9H-J). They efficiently entered CMs under H_2_O_2_ stimulation, colocalizing with mitochondria, and subsequently scavenged excess mitochondrial ROS (Figure 9K). This mitochondrial stabilization by SSSe NPs significantly reduced oxidative stress-induced apoptosis *in vivo* (Figure 9L). This strategy not only offers a promising treatment option for MI but also provides inspiration for other ischemic diseases 141.

To summarize, the use of inorganic nanomedicines, while promising, must consider the potential for long-term toxicity due to metal accumulation in tissues. Developing biodegradable or bioresorbable variants of these nanomaterials could mitigate these concerns. Furthermore, exploring the combination of inorganic nanozymes with organic polymers or natural carriers might offer a balance between high catalytic activity and enhanced biocompatibility. Researchers should also investigate the use of these nanozymes in conjunction with advanced imaging techniques to monitor real-time treatment efficacy and dynamically adjust therapeutic strategies.

### 6.5. Gas-supplying nanomedicines

Gas therapy, including the use of therapeutic gases such as O_2_, NO, hydrogen sulfide (H_2_S), and H_2_, has emerged as a novel approach for treating various cardiovascular diseases 142. However, the clinical application of gas therapy faces significant challenges due to the inherent difficulties in controlling the delivery, dosage, and stability of these gaseous molecules. Nanocarriers offer a transformative solution by addressing these limitations and enhancing the therapeutic efficacy of gas therapy.

O_2_ therapy has been extensively investigated for enhancing cell survival in the ischemic myocardium by elevating O_2_ levels in infarcted tissues and blood 143. Exogenous O_2_ sustains the viability and metabolism of cardiac cells during acute ischemia, with the potential to mitigate infarct size and decrease the likelihood of lethal arrhythmias. Hemoglobin, a tetrameric protein comprising two α- and two β-polypeptide chains, each containing an iron-containing heme group capable of binding a single O_2_, has been utilized as an O_2_ carrier. Li *et al.* demonstrated that polymerized placenta hemoglobin (PolyPHb) safeguarded isolated hearts from I/RI by alleviating of NO-mediated myocardial apoptosis and the restoration of nitroso-redox balance 144. Compared to PolyHb, PEG-conjugated hemoglobin (PEG-Hb) possesses a larger molecular radius, making it less prone to extravasation or the induction of hypertension. Additionally, surface conjugation with PEG reduces immunogenicity and increases plasma viscosity, rendering it a more optimized O_2_ carrier. The carbon monoxide form of PEGylated hemoglobin has been shown to reduce infarct size when administered either before left anterior descending coronary artery (LAD) occlusion or during reperfusion 142.

**Figure 10:**
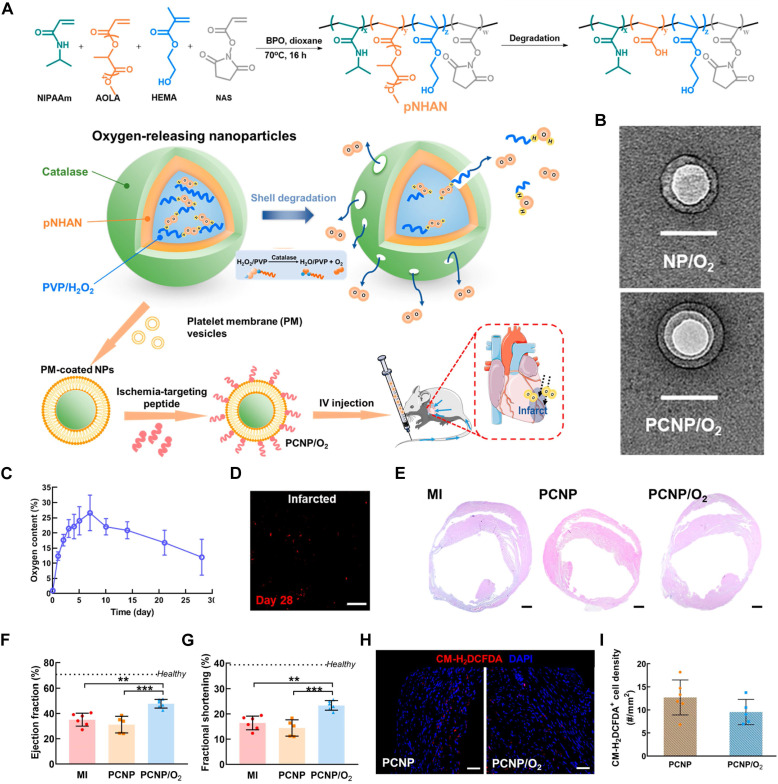
(A) Schematic illustration of the design of PCNP/O_2_. (B) TEM images of NP/O_2_ and PCNP/O_2_. (C) Oxygen release kinetics of PCNP/O_2_ (D) Fluorescent images of the infarcted regions of the myocardium. (E) Representative images of Masson's trichrome staining. (F-G) Cardiac functional measurement including EF% and FS%. (H-I) Representative images and quantification of dihydroethidium staining. Adapted with permission from 52, copyright 2022 John Wiley and Sons.

Although artificial O_2_ carriers utilizing hemoglobin have demonstrated certain preclinical advantages, these systems fail to achieve sustained O_2_ release, a crucial factor for cell survival before the establishment of angiogenesis. To address the limitations in O_2_ delivery for treating IHD, Guan *et al.* developed O_2_-generating microspheres capable of gradual O_2_ release. These NPs were engineered with a degradable polymer shell and a core composed of a polyvinylpyrrolidone (PVP)/H_2_O_2_ complex (PCNP/O_2_) (Figure 10A-B). Under conditions simulating infarcted cardiac tissue with 1% O_2_, the NPs exhibited sustained O_2_ release for 4 weeks (Figure 10C), with the O_2_ concentration reaching approximately 12% within 24 hours. PCNP/O_2_ NPs were retained in the infarcted heart for up to 28 days (Figure 10D). These results indicate that PCNP/O_2_ NPs have prolonged retention in ischemic heart tissue and provide sustained O_2_ delivery to the surrounding environment. After 4 weeks of treatment with PCNP/O_2_ NPs, the infarct size was reduced compared to the MI and PCNP groups (Figure 10E). Additionally, the PCNP/O_2_ group exhibited a significantly higher left ventricular ejection fraction (EF%) and fractional shortening (FS%) (Figure 10F-G). Moreover, the released O_2_ did not induce oxidative stress in the infarcted myocardium (Figure 10H-I) 52.

NO has been demonstrated in numerous experimental studies to modulate IHD 145, 146. Administration of NOS inhibitors has been reported to exacerbate myocardial necrosis, supporting the concept that endogenous NO confers protection against MI/RI 147. Abundant evidence suggests that supplemental exogenous NO is efficacious against MI/RI through multifaceted mechanisms, including the regulation of vasomotor tone and angiogenesis, reduction of myocardial ROS production, and inhibition of apoptosis, among others 148. A platelet membrane-coated NP (B-P@PLT) was developed, featuring a polymeric core loaded with BNN6, an ultrasound-responsive NO donor, designed for the targeted treatment of MI/RI. B-P@PLTs can release NO during ultrasound irradiation, safeguarding CMs both *in vitro* and *in vivo* by reducing ROS and promoting vascular regeneration 149.

Additionally, Wang *et al.* developed a novel GSH-activated, water-dispersible, slow, and controllable H_2_S release system (DATS-MSN) utilizing diallyl trisulfide (DATS) and MSNs. DATS-MSNs exhibit a significantly slower process of H_2_S generation, thereby demonstrating superior cardioprotective effects in MI/R models by enhancing antioxidant enzyme activities 150. Hydrogen gas (H_2_), a well-known molecule, has also been proposed for its potential in preventing and treating organ dysfunction induced by MI/RI. Zheng *et al.* developed an ultrasound-visible H_2_ delivery system by encapsulating H_2_ inside microbubbles (H_2_-MBs). This system enables a significant increase in the concentration of H_2_ in a unit volume under normal temperature and pressure conditions. H_2_-MBs can be visually tracked using ultrasound imaging systems and can effectively release therapeutic gas. *In vivo* results demonstrated that this approach markedly attenuated CM apoptosis and reduced myocardial inflammation and oxidative damage in MI/RI rats 151.

In summary, for gas-supplying nanomedicines, future research could explore the use of smart responsive materials that modulate gas release in response to specific physiological signals such as pH, temperature, or enzyme activity, thereby enhancing the precision of therapy. Additionally, combining gas therapy with other therapeutic agents, such as angiogenic factors or anti-apoptotic drugs, could create a synergistic effect, improving the overall outcome. Evaluating these systems in larger animal models and early-phase clinical trials will be critical for understanding their safety, efficacy, and potential for human application.

### 6.6. Other antioxidant nanomedicines

Cyclosporine A (CsA) is a cyclic polypeptide consisting of 11 amino acids 152. It inhibits the excessive opening of the mPTP by binding to cyclophilin D on the inner mitochondrial membrane, thereby suppressing the release of ROS from mitochondria and CM apoptosis 153. However, CsA exhibits poor solubility in water, and its natural distribution *in vivo* lacks myocardial targeting. To address this issue, CsA was encapsulated with Poly (5,5-dimethyl-4,6-dithio-propylene glycol azelate) (PTK) to form CsA@PTK, which was further enveloped with platelet membranes to create a Treg biomimetic NP (CsA@PPTK). Experimental results indicated that CsA@PPTK actively accumulated in the ischemic myocardium of MI/RI mice and effectively scavenged ROS. The construction of these biomimetic NPs has a significant therapeutic effect on MI/RI and holds great potential for application in MI/RI treatment 154.

ONO-1301, a novel drug, is a synthetic prostacyclin IP receptor agonist with atypical prostanoid structures 155. This unique feature enhances the biological and chemical stability of the compound, leading to prolonged prostacyclin activity *in vivo*. Studies have demonstrated that ONO-1301 binds to IP receptors on endothelial cells, vascular smooth muscle cells, or fibroblasts, triggering the release of protective cytokines. Encapsulation of ONO-1301 into PEGylated liposomes prolongs its circulation time in the bloodstream and enhances its accumulation within cardiac tissue. Results showed that intravenously injected ONO-1301-containing NPs (ONO-1301NPs) selectively accumulate in the injured myocardium of rats. Consequently, rats injected with ONO-1301NPs exhibited a smaller infarct size, better-preserved capillary networks, and improved MBF 156. Additionally, a novel H_2_O_2_-responsive antioxidant copolyoxalate, composed of hydroxybenzyl alcohol (HBA) (HPOX) and vanillyl alcohol (VA) (PVAX), has been developed for antioxidant therapy in MI/RI. PVAX and HPOX were synthesized using naturally occurring compounds with intrinsic antioxidant and anti-inflammatory properties, VA and HBA, respectively. These antioxidant polymeric prodrug NPs can be rapidly activated by H_2_O_2_ at the site of ROS generation, mitigating I/R-induced injuries through antioxidant, anti-inflammatory, and antiapoptotic mechanisms 157.

Moreover, maintenance intracellular oxidative stress necessitates the involvement of multiple signaling molecules. Modulating cellular signaling pathways through small interfering RNA (siRNA) offers a method for intervening in oxidative stress. Researchers have proposed a novel approach using platelet membrane-camouflaged PLGA NPs (PMVs@PLGA complexes) for the systemic delivery of miRNA inhibitors. By targeting the Nrf2 regulatory pathway, these NPs can potentially enhance cardiac resilience against MI/RI 51. Ji *et al.* encapsulated microRNA-146a (miR-146a) into milk exosomes (MEs) to create MEs-miR-146a, which can be administered both orally and intravenously. MEs-miR-146a exerts significant antiapoptotic and anti-inflammatory effects by inhibiting the IRAK1/TRAF6/NF-κB signaling pathway 158. For future research, combining traditional antioxidant therapies with cutting-edge molecular technologies, such as CRISPR/Cas9 for gene editing or RNA interference, could offer novel therapeutic avenues. Exploring the potential of integrating diagnostic capabilities into these therapeutic platforms could enable real-time monitoring of disease progression and treatment efficacy, leading to more personalized and adaptive therapeutic strategies. Furthermore, considering the scalability of these advanced nanomedicines and their integration into existing clinical frameworks will be crucial for their successful translation into clinical practice.

## 7. Conclusion and future outlook

This article thoroughly reviews advancements in nanomedicines for addressing IHD. Understanding the pathological mechanisms of IHD is essential for the strategic selection of therapeutics. This review progresses by evaluating antioxidant mechanisms and drug molecules through both *in vitro* and *in vivo* studies. Attention is then given to the advent of nanocarriers that improve drug delivery, focusing particularly on their role in enhancing antioxidant nanomedicines for treating IHD. These nanocarriers overcome traditional drug limitations by increasing drug localization at the target site, reducing the overall dosage, and minimizing side effects, thereby markedly enhancing treatment efficacy and safety. This review concludes with a systematic summary of delivery technologies for various therapeutic agents aimed at IHD. Our review uniquely narrows down to antioxidant nanomedicines, providing an in-depth exploration of their design, targeted delivery mechanisms, and multifunctional nanomaterials. By specifically focusing on MI and MI/RI, we offer detailed discussions of their pathophysiology and targeted treatment strategies. We highlight cutting-edge nanocarrier systems, including innovative gas therapy-based nanocarriers, and provide clear categorization of antioxidant nanomedicines, enhancing logical flow and reader comprehension. Additionally, we integrate interdisciplinary insights from materials science, nanotechnology, and cardiology, presenting a holistic view and underscoring the collaborative efforts required to advance this field.

Despite significant strides in the development of cardioprotective drugs and cellular therapies for IHD 78, 85, 159, the efficacy of these interventions remains largely limited to preclinical studies. ROS have been identified as primary contributors to cellular damage under both hypoxic and reoxygenation conditions 6, 25, yet effective interventions remain elusive. Our comprehensive analysis addresses several critical factors: initially, there are considerable differences between commonly used animal models in preclinical studies and the human physiological context. Many studies utilize young, healthy animals, which can skew results due to their superior regenerative capabilities compared to the typically older, multimorbid human cardiac patients observed in clinical environments. Additionally, human research is plagued by numerous confounding variables, such as diet, sex, ethnicity, and psychological factors, which significantly complicate the interpretation of results. Moreover, the intricate homeostatic mechanisms in humans necessitate a multifaceted approach to therapy, targeting multiple pathways rather than a single molecular target. Finally, the current clinical use of these drugs is limited by their narrow antioxidant scope and inherent toxicity, which restricts their feasible dosage and thus their observable therapeutic impacts.

Can nanomedicines represent a significant advance over traditional pharmaceuticals in therapeutic efficacy? Theoretically, engineered carriers offer promising improvements in drug stability and targeted delivery. These nanocarriers, categorized by their carrier materials, exhibit unique properties that cater to the diverse requirements of therapeutic agents. PNPs, such as those made from PLGA and PEG, have shown remarkable capabilities in stabilizing and prolonging the circulation time of enzymatic antioxidants like SOD and CAT 113. By encapsulating these enzymes, PNPs protect them from degradation, ensuring a sustained therapeutic effect in mitigating oxidative stress. Similarly, liposomes have emerged as versatile carriers for various antioxidants, such as curcumin and RSV 108, by enhancing their solubility and bioavailability. The amphiphilic nature of liposomes facilitates the incorporation of hydrophobic small molecules, providing a controlled-release profile and targeted delivery to ischemic myocardial tissues.

In the realm of inorganic nanomedicines, MOFs and Ce NPs have gained attention for their intrinsic catalytic activities that mimic natural antioxidant enzymes. MOFs, with their high surface area and tunable porosity, effectively deliver SOD-mimetic and CAT-mimetic agents, offering a robust defense against ROS 112. Ce NPs, known for their redox cycling between Ce^3+^ and Ce^4+^, exhibit multi-enzyme activities, scavenging a wide range of ROS and thus protecting cardiac tissues from oxidative damage 139. Moreover, GGNs, which release therapeutic gases like H_2_S and NO, have shown promise in modulating oxidative stress and inflammatory responses. These nanocarriers ensure the controlled and sustained release of gases, targeting the ischemic myocardium and enhancing the therapeutic outcomes 150.

The development of MSNs and NLCs has further advanced the delivery of small molecule drugs. MSNs, with their large surface area and customizable pore sizes, enable high drug loading and controlled release of antioxidants like que and BN 62. NLCs, combining solid and liquid lipids, offer improved drug encapsulation efficiency and stability, prolonging the retention time of therapeutic agents in the bloodstream 108. These innovative nanocarrier systems not only enhance the pharmacokinetic profiles of antioxidant therapies but also improve their targeting capabilities, thereby maximizing their efficacy in treating IHD. Therefore, these advanced nanotechnologies undoubtedly have significant effects and broad prospects for improving antioxidant drugs.

However, the field of nanomedicine faces numerous unresolved challenges in both research and practical applications. For instance, the introduction of biomaterials may induce unknown biological toxicity and risks 160. Additionally, achieving specific functionalities often requires complex modifications, which introduce additional exogenous substances into the organism and potentially increase the physiological burden. Furthermore, the biological processes of many biomaterials within the body remain poorly understood, presenting significant challenges to their safe and effective use. Despite enhancements in drug performance, current scientific techniques fall short of achieving optimal therapeutic effects, highlighting the gap between current capabilities and ideal outcomes. Ultimately, while nanomedicines have facilitated incremental improvements in certain areas, they have not fundamentally changed the paradigm of disease treatment research. To achieve substantial progress, a deeper understanding of the complex composition and regulatory mechanisms of biological systems is essential.

Nanomedicines primarily act as carriers for antioxidant drugs, enhancing their delivery and efficacy. However, it is important to recognize that certain nanocarriers themselves possess inherent antioxidant properties and contribute directly to therapeutic effects. For instance, nanozymes like Ce oxide NPs exhibit intrinsic antioxidant activities, neutralizing ROS independently of any encapsulated drugs 161. Additionally, nanocarriers such as PDA NPs also exhibit intrinsic antioxidant properties, offering a dual function as both carriers and active agents in mitigating oxidative stress 106. These examples underscore the dual role of specific nanocarriers in antioxidant therapy, functioning both as carriers and active therapeutic agents. This dual functionality enhances the therapeutic potential of nanomedicines, offering a more comprehensive approach to treating IHD.

What should be the focus of our future research efforts? Initially, enhancing our understanding of the pathophysiology of diseases is crucial, as it allows for the identification of truly effective intervention targets that could lead to substantial breakthroughs. The challenges in treating IHD extend beyond oxidative stress to include autophagy and apoptosis dysregulation, endothelial dysfunction, ER stress, ischemia-induced angiogenesis, metabolic dysregulation, and fibrosis, all of which form the basis for current design strategies of nanomedicines 162. These factors highlight the complexity of IHD and the necessity for comprehensive approaches that address multiple aspects of the disease. Although this review focuses specifically on the challenges associated with oxidative stress, it is essential to consider the broader array of issues in future research to develop more effective therapeutic strategies. By tackling these multifaceted challenges, we can advance the field of nanomedicine and improve outcomes for patients suffering from IHD.

Furthermore, the development of novel biomaterials needs to be advanced, prioritizing materials that can be effective without complex modifications. This will simplify their application in therapeutic interventions. Finally, a comprehensive understanding of the pathways, interference factors, and metabolic processes of biomaterials within the body is essential for their successful clinical translation.

## Figures and Tables

**Table 1:** Some of the major endogenous antioxidants and their sites of action in CMs.

Antioxidant	Site of Action	Action
SOD	Cytoplasm, mitochondria, extracellular space	2•O_2_^-^ + 2H^+^ → H_2_O_2_ + O_2_
CAT	Peroxisomes, mitochondrial membrane	H_2_O_2_→2H_2_O+O_2_
GPx	Cytoplasm, mitochondria, nucleus	H_2_O_2_+2GSH→2H_2_O+GSSG
GSH	Cytoplasm, mitochondria, nucleus	GSSG + NADPH → 2GSH + NADP^+^
Thioredoxin	Cytoplasm, nucleus, cell membrane, mitochondria, extracellular space	2Trx-SH ↔ Trx-S-S-Trx
HSPs	Cytoplasm, nucleus	Various chaperone and antioxidant functions
α-tocopherol	Cell membrane	Break lipid peroxidation chain and LDL reaction
Vitamin C (ascorbic acid)	Cytoplasm, extracellular space	Ascorbate + •ROO → Dehydroascorbate + •RO + H_2_O
CoQ10	Mitochondria	CoQ10 + •ROO → CoQ10H + •RO
Metallothioneins	Cytoplasm, nucleus	MT-SH + ROS → MT-S-S-MT + reduced ROS
Bilirubin	Cytoplasm, extracellular space	Various antioxidant and anti-inflammatory functions
β-Carotene (pro-vitamin A)	Plasma	Inhibits oxidation of LDL

**Table 2:** Diagnostic Nanomedicines for IHD.

Imaging modality	Nanomedicine type	Advantage	Model	Ref.
MRI	SPIONs	High spatial, temporal resolution, high soft tissue contrast, the ability of quantitative imaging	MI	36
	Hsp70-SPION		MI	163
	Gd-CDs		MI/RI	97
	MnO-OA		MI	98
CT	CNA35	Easily available, rapid imaging, high image quality, noninvasive	MI	100
PET	Na[^18^F]F		MI/RI	101
	^68^Ga^3+^		MI	164
Fluorescence imaging	CD47-EVs	Noninvasive, targeting of multiple biological factors, high sensitivity	MI/RI	103
	SiO2@pDA-DNA-CeO2		MI/RI	104
	GF/TPO		MI/RI	105

**Table 3:** The strategies, including achievements and limitations in targeted delivery of nanomedicines in reducing oxidative stress.

Phase of cascade	Type of materials	Cargos	Achievement	Limitation	Model	Administration	Ref.
**Enzymatic antioxidants**	PEG-PBD/PEG-PPO	SOD	•O_2_^-^ → H_2_O_2_	Short half-life and limited stability of SOD	MI/R	Intramyocardial	113
	PCADK	SOD1	•O_2_^-^ → H_2_O_2_	Inadequate concentration	MI/R	Intramyocardial	107
	ZrMOF	SOD	•O_2_^-^ → H_2_O_2_	Potential toxicity and long-term stability	MI	Intramyocardial	112
**Nonenzymatic antioxidants**	PEG-allomelanin	Allomelanin	Antioxidant and anti-inflammatory	Poor solubility and potential immunogenicity	MI	Tail vein	120
	EVs	Mel	Scavenging ROS	Difficulty in controlling the release and bioavailability	MI	Intramyocardial	121
	PEG-bilirubin	Bilirubin	Antioxidant	Poor stability and potential toxicity	MI/R	Intraperitoneal	24
	PEG-PDA	PDA	Scavenging •O_2_^-^ and •OH and alleviating Fe^2+^ accumulation	Long-term toxicity	MI/R	Tail vein	106
	Macrophage membrane-coated PDA	PDA	Scavenging •O_2_^-^ and •OH	Complexity of manufacturing	MI/R	Tail vein	117
	Liposome	EGCG and CoQ10	Eradicating ROS and mitigating apoptosis	Potential degradation of encapsulated drugs	MI	Tail vein	71
	Liposome	CoQ10	Antioxidant	Potential for drug leakage and limited precise targeting	MI	Coronary infusion	53
	PLGA	LA	Reducing oxidative stress, senescence, DNA damage, cytokine-related processes, apoptosis, and ferroptosis	Slow and incomplete release of drugs	MI	Hydrogel delivery	127
**NSMs**	MSN	Que	Inhibiting cell apoptosis and oxidative stress	Poor bioavailability and rapid metabolism	MI/R	Intravenous	62
	PLGA	Que	Antioxidant	Rapid clearance and limited targeting efficiency	MI/R	/	63
	ZIF-8 cored QSF@Z-NCs	Que	Reducing apoptosis and promoting regeneration	Complex production and safety concerns	MI	Intramyocardial	165
	MOF	Que	Antioxidant and anti-inflammatory	Long-term toxicity and limited targeting efficiency	MI	Tail vein	38
	Solid lipid NPs	PUE	Antioxidant	Limited drug release control	MI	Intravenous	129
	PEG-SLNs	BN	Antioxidant	Poor stability and potential for drug leakage	MI	Intraperitoneal	108
	Hydrogel	EGCG and Rhein	Antioxidant and anti-inflammatory	Complexity of application and long-term efficacy concerns	MI/R	Intramyocardial	166
	EVs	Curcumin and miR-144-3p	Antioxidant and inhibiting of apoptosis	Limited targeting specificity and Complex production	MI	Tail vein	167
	PGMA	Curcumin	Antioxidant	Limited targeting precision and potential toxicity	MI/R	Coronary artery perfusion	168
	EVs	Curcumin	Antioxidant	Limited drug loading capacity and challenges in large-scale production	MI	Intravenous	131
	β-MEND	RSV	Maximized cell respiration	Limited mitochondrial targeting efficiency	/	/	169
	mPEG-b-O (D, L-Leu)	RSV	Inhibiting of apoptosis	Limited bioavailability and potential aggregation	MI/R	Subcutaneous	170
	IMTP-ployHis-PEG3400-hyd-PLGA/ SS-31-PEG8-PLGA	RSV	Antioxidant	Limited targeting accuracy and complex delivery mechanism	MI/R	Intravenous	78
	PLGA	RSV	Antioxidant and anti-inflammatory	Limited drug release control	MI	per oral	77
	Lipid-polymer hybrid NPs	Salvianolic acid B and PNS	Enhancing targeted drug delivery efficiency	Limited receptor targeting efficiency	MI	Tail vein	76
	MSN	Salvianolic acid B	Inhibiting of oxidative stress and apoptosis	Potential for limited drug loading capacity	MI/R	Ingastric administration	171
	PEG-b-PPS	Rg3	Inhibiting oxidative stress, inflammation, and fibrosis promotion	Limited ROS responsiveness	MI/R	Intramyocardial	132
	Silica NPs	Notoginsenoside R1	Inhibiting oxidative stress, inflammation, and apoptosis	Limited targeting efficiency and potential off-target	MI	Tail vein	84
	mPEG-PLGA	Panax notoginseng	Antioxidant	Limited long-term stability	MI/R	Orally	130
	PEG-SLNs	Sch B	Reducing the infarction size	Limited MMP sensitivity	MI	Tail vein	73
	mPEG-PLA-TPGS	Tanshinone IIA	Reducing inflammation, apoptosis, and fibrosis	Limited specificity	MI	Intravenous	80
**Inorganic nanoenzymes**	CVNRs	/	SOD-like activities	Limited efficacy	MI/R	Intravenous	138
	Pd@CeO_2_	/	CAT- and SOD-like activities	Complexity manufacturing and uncertain long-term biocompatibility	MI/R	Intravenous	35
	TA-Ce	/	CAT- and SOD-like activities	Limited targeting specificity	MI/R	Intravenous	161
	Cu-TCPP-Mn	/	CAT- and SOD-like activities	Potential instability and limited ROS scavenging efficiency	MI	Intravenous	172
	RuO_2_@BSA	/	CAT- and SOD-like activities	Potential cytotoxicity	MI/R	Intravenous	173
	ZIF-8	/	CAT- and SOD-like activities	Potential for incomplete ROS scavenging	MI	In situ delivery	174
	Mn_3_O_4_@PDA	MSCs	CAT- and SOD-like activities	Potential for limited MRI tracking sensitivity	MI	Intravenous	140
	CuCe	/	CAT- and SOD-like activities	Uncertain long-term biocompatibility	MI	Intramyocardial	139
	Au@Pt	/	CAT- and SOD-like activities	Limited long-term stability	MI	Intramyocardial	175
	Au@Se	L-Arg	CAT- and SOD-like activities	Limited targeting specificity	MI/R	Intravenous	176
	SSSe	/	CAT- and SOD-like activities	Limited long-term stability of the self-sustaining antioxidant system	MI	Intravenous	141
	AS-I/SNCs	SS31	CAT-, SOD-, and GPx-like activities	Limited targeting specificity	MI/R	Intravenous	83
	ZIF-8zyme	/	CAT-, SOD-, and GPx-like activities	Limited targeting efficiency	MI	/	177
	Fe-Cur@TA	Curcumin	CAT-, SOD-, and POD-like activities	Limited targeting efficiency	MI	Intravenous	178
	MnO2 Fenozymes	/	CAT-, SOD-, and POD-like activities	Limited mitochondrial targeting efficiency	MI/R	Hydrogel delivery	179
	PtsaN-C	/	CAT-, SOD-, and POD-like activities	Limited targeting specificity	MI/R	Intramyocardial	180
**Gas-generating nanomedicine**	PolyPHb	Hemoglobin	Elevating SOD activity and preserving mitochondrial ATP synthesis	Limited long-term efficacy	MI/R	/	144
	PEGy-Hb	Hemoglobin	Reducing infarct size	Limited targeting specificity	MI/R	Intraperitoneal	142
	PCNP/O_2_	/	Enhancing cardiac cell survival, stimulating, angiogenesis, and suppressing fibrosis	Limited Long-Term Efficacy and safety concerns	MI	Intravenous	52
	PUAO-CPO-Collagen	Ca^2+^ peroxide	Reducing scar formation, attenuating adverse cardiac remodelling and decreasing oxidative stress	Limited *in vivo* testing	MI	Intravenous	85
	SOD/PAC	G-CSF, NO/H_2_S	Reducing ROS, inflammation level and relieving Ca^2+^ overload	Limited targeted delivery efficiency	MI/R	Intravenous	181
	B-P@PLT	BNN6	Promoting angiogenesis, reducing ROS production	Challenges in controlled release	MI/R	Intravenous	149
	Chitosan hydrogel	NO	Antioxidant	Uncertain biodegradability and biocompatibility	MI/R	Intramyocardial	182
	DATS-MSN	H_2_S	Inhibiting oxidative stress and inflammation	Delayed response due to slow release	MI/R	Tail vein	150
	Pluronic F-127/KAT	Keratin and H_2_S	Ameliorating microvascular obstruction, preventing myocardial fibrosis, and attenuating cardiac inflammation	Unclear long-term safety	MI/R	Myocardial surface	183
	C_3_F_8_-loaded microbubbles	H_2_S	Inhibiting oxidative stress and inflammation	Inconsistent dosage delivery	MI/R	Tail vein	184
	Microbubble	H_2_	Antioxidant	Limited precision in targeted Delivery	MI/R	Tail vein	151
**Others**	PEG-SLNs	ONO-1301	Anti-inflammatory	Complex production process	MI/R	Intravenous	156
	PTK	CsA	Scavenging ROS	Unclear relevance to chronic inflammation	MI/R	Tail vein	154
	PLGA	CsA	Inhibiting mPTP opening	Limited efficiency in targeted delivery	MI/R	Intravenous	185
	Platelet Membrane-Encapsulated MSN	SS31	Scavenging ROS	Potential immune response	MI/R	Tail vein	186
	PLGA-TK-PEG/ HA-Diol-HYD	SS31/CsA	Scavenging ROS	Uncertain long-term effects	MI/R	Intramyocardial	79
	PLL-PEG-PLL	Exenatide	Attenuating the oxidative stress	Uncertain drug stability	MI/R	Subcutaneous	187
	PGMA	Alpha-interacting domain (AID)	Reducing in release of creatine kinase and lactate dehydrogenase	Insufficient long-term efficacy	MI/R	Perfusion on Langendorff's apparatus	188
	HPOX/PVAX	HBA/VA	Antioxidant	Potential toxicity	MI/R	Intramyocardial	157
	Platelet membrane-coated PLGA	microRNA	Inhibiting of ROS and apoptosis	Variable targeting efficiency	MI/R	Tail vein	51
	EVs	microRNA	Inhibiting apoptosis and inflammatory	Potential immune response	MI/R	Tail vein	158
	DNA nanostructures	/	Reducing the ROS production	Uncertain long-term biocompatibility	MI/R	/	189

## Abbreviations

IHD: Ischemic heart disease
MI: Myocardial infarction
ROS: Reactive oxygen species
Mel: Melatonin
HF: Heart failure
WHO: World Health Organization
PCI: Percutaneous coronary intervention
MI/RI: Myocardial ischemia/reperfusion injury
EVs: Extracellular vesicles
ETC: Electron transport chain
hNVs: Hybrid nanovesicles
NOX: NADPH oxidases
PKC: Protein kinase C
NF-κB: Nuclear factor-kappa B
eNOS: Nitric oxide synthase
DAMPs: Damage-associated molecular patterns
NO: Nitric oxide
•O_2_^-^: Superoxide anions
•OH: Hydroxyl radicals
H_2_O_2_: Hydrogen peroxide
GPx: Glutathione peroxidase
CM: Cardiomyocyte
PRRs: Pattern recognition receptors
TLRs: Toll-like receptors
NLRs: Nucleotide-binding oligomerization domain-like receptors
MAPKs: Mitogen-activated protein kinase
AP-1: Activator protein-1
TCA: Tricarboxylic acid
HMGB-1: High mobility group box-1
HSPs: Heat shock proteins
TNF-α: Tumor necrosis factor-alpha
IL-1β: Interleukin 1-beta
IL-6: Interleukin-6
IL-18: Interleukin-18
SR: Sarcoplasmic reticulum
SERCA: Sarcoplasmic/endoplasmic reticulum Ca^2+^-ATPase
PMCA: Plasma membrane Ca²⁺-ATPase
SOD: Superoxide dismutase
GSH: Glutathione
CAT: Catalase
PDA: Polydopamine
NAs: Nucleic acids
GGNs: Gas-generating nanoplatforms
CoQ10: Coenzyme Q10
NSMs: Natural small molecule drugs
HAT: Hydrogen atom transfer
BHA: Butylated hydroxyanisole
BHT: Butylated hydroxytoluene
TBHQ: Tertiary butylhydroquinone
PG: Propyl gallate
PNPs: Polymeric NPs
PEG: Polyethylene glycol
PLA: Poly (lactic acid)
PLGA: Poly (lactic-co-glycolic acid)
MSCs: Mesenchymal stem cells
RBCs: Red blood cells
hEPs: Human embryonic stem cell-derived epicardial cells
MRI: Magnetic resonance imaging
CT: Computed tomography
PET: Positron emission tomography
SPIONs: Superparamagnetic iron oxide NPs
USPIOs: Ultrasmall superparamagnetic iron oxide NPs
Gd-CDs: Gadolinium-doped carbon dots
CAD: Coronary artery disease
MBF: Myocardial blood flow
MFR: Myocardial flow reserve
ICG: Indocyanine green
Mn: Manganese
MOF: Metal-organic frameworks
1,8-DHN: 1,8-dihydroxynaphthalene
ADSCs: Adipose-derived stem cells
LA: Alpha-lipoic acid
MSNs: Mesoporous silica NPs
PUE: Puerarin
RSV: Resveratrol
Que: Quercetin
BN: Baicalin
AUC: Area under the curve
Sch B: Schisandrin B
SLNs: Solid lipid NPs
MMP: Matrix metalloproteinase
PNS: Panax notoginsenoside
IMTP: Ischemic myocardial-targeted peptide
Rg3: Ginsenoside Rg3
PPS: Poly (propylene sulfide)
Ce: Cerium
CVNRs: Ce vanadate nanorods
H_2_S: Hydrogen sulfide
HCMECs: Human cardiac microvascular endothelial cells
PolyPHb: Polymerized placenta hemoglobin
LAD: Left anterior descending coronary artery
PVP: Polyvinylpyrrolidone
DATS: Diallyl trisulfide
mPTP: Mitochondrial permeability transition pore
EF%: Ejection fraction
FS%: Fractional shortening
H_2_: Hydrogen gas
CsA: Cyclosporine A
PTK: Poly (5,5-dimethyl-4,6-dithio-propylene glycol azelate)
HBA: Hydroxybenzyl alcohol
VA: Vanillyl alcohol
SiRNA: Small interfering RNA

## References

[1] Berry C, Corcoran D, Hennigan B, Watkins S, Layland J, Oldroyd KG (2015). Fractional flow reserve-guided management in stable coronary disease and acute myocardial infarction: recent developments. Eur Heart J.

[2] Collaborators GBDCoD (2018). Global, regional, and national age-sex-specific mortality for 282 causes of death in 195 countries and territories, 1980-2017: a systematic analysis for the Global Burden of Disease Study 2017. Lancet.

[3] Shi H, Huang Z, Xu T, Sun A, Ge J (2022). New diagnostic and therapeutic strategies for myocardial infarction via nanomaterials. EBioMedicine.

[4] Xiao H, Zhang M, Wu H, Wu J, Hu X, Pei X (2022). CIRKIL exacerbates cardiac ischemia/reperfusion injury by interacting with Ku70. Circ Res.

[5] Zhai M, Li B, Duan W, Jing L, Zhang B, Zhang M (2017). Melatonin ameliorates myocardial ischemia reperfusion injury through SIRT3-dependent regulation of oxidative stress and apoptosis. J Pineal Res.

[6] Bugger H, Pfeil K (2020). Mitochondrial ROS in myocardial ischemia reperfusion and remodeling. Biochim Biophys Acta Mol Basis Dis.

[7] Wang L, Ma Q (2018). Clinical benefits and pharmacology of scutellarin: A comprehensive review. Pharmacol Ther.

[8] Mubagwa K, Flameng W (2001). Adenosine, adenosine receptors and myocardial protection: an updated overview. Cardiovasc Res.

[9] Ma W, Guo W, Shang F, Li Y, Li W, Liu J (2020). Bakuchiol alleviates hyperglycemia-induced diabetic cardiomyopathy by reducing myocardial oxidative stress via activating the SIRT1/Nrf2 signaling pathway. Oxid Med Cell Longev.

[10] Zhang Y, Wang Y, Xu J, Tian F, Hu S, Chen Y (2019). Melatonin attenuates myocardial ischemia-reperfusion injury via improving mitochondrial fusion/mitophagy and activating the AMPK-OPA1 signaling pathways. J Pineal Res.

[11] Zhang Z, Dalan R, Hu Z, Wang J-W, Chew NWS, Poh K-K (2022). Reactive oxygen species scavenging nanomedicine for the treatment of ischemic heart disease. Advanced Matererials.

[12] Patra JK, Das G, Fraceto LF, Campos EVR, Rodriguez-Torres MDP, Acosta-Torres LS (2018). Nano based drug delivery systems: recent developments and future prospects. J Nanobiotechnology.

[13] Jo DH, Kim JH, Lee TG, Kim JH (2015). Size, surface charge, and shape determine therapeutic effects of nanoparticles on brain and retinal diseases. Nanomedicine.

[14] Zhao D, Huang X, Zhang Z, Ding J, Cui YC, Chen X (2021). Engineered nanomedicines for tumor vasculature blockade therapy. Wiley Interdiscip Rev Nanomed Nanobiotechnol.

[15] Lakshmi BA, Kim S (2019). Current and emerging applications of nanostructured metal-organic frameworks in cancer-targeted theranostics. Mater Sci Eng C.

[16] Libby P, Theroux P (2005). Pathophysiology of coronary artery disease. Circulation.

[17] Bruning RS, Sturek M (2015). Benefits of exercise training on coronary blood flow in coronary artery disease patients. Prog Cardiovasc Dis.

[18] Joshi PH, Nasir K (2015). Discordance between risk factors and coronary artery calcium: Implications for guiding treatment strategies in primary prevention settings. Prog Cardiovasc Dis.

[19] Vogel B, Claessen BE, Arnold SV, Chan D, Cohen DJ, Giannitsis E (2019). ST-segment elevation myocardial infarction. Nat Rev Dis Primers.

[20] Wendelboe AM, Raskob GE (2016). Global burden of thrombosis epidemiologic aspects. Circ Res.

[21] PenaDuque MA, RomeroIbarra JL, GaxiolaMacias MBA, AriasSanchez EA (2015). Coronary atherosclerosis and interventional cardiology. Arch Med Res.

[22] Charnock JS (1994). Lipids and Cardiac-Arrhythmia. Progress in Lipid Research.

[23] Bentzon JF, Otsuka F, Virmani R, Falk E (2014). Mechanisms of plaque formation and rupture. Circ Res.

[24] Ai W, Bae S, Ke Q, Su S, Li R, Chen Y (2021). Bilirubin nanoparticles protect against cardiac ischemia/reperfusion injury in mice. J Am Heart Assoc.

[25] D'Autreaux B, Toledano MB (2007). ROS as signalling molecules: mechanisms that generate specificity in ROS homeostasis. Nat Rev Mol Cell Bio.

[26] Jang S, Lewis TS, Powers C, Khuchua Z, Baines CP, Wipf P (2017). Elucidating mitochondrial electron transport chain supercomplexes in the heart during ischemia-reperfusion. Antioxid Redox Signal.

[27] Zhao H, Zhang R, Yan X, Fan K (2021). Superoxide dismutase nanozymes: an emerging star for anti-oxidation. J Mater Chem B.

[28] Bokare AD, Choi W (2014). Review of iron-free Fenton-like systems for activating H2O2 in advanced oxidation processes. J Hazard Mater.

[29] Lambeth JD, Krause KH, Clark RA (2008). NOX enzymes as novel targets for drug development. Semin Immunopathol.

[30] Brandes RP, Weissmann N, Schröder K (2014). Redox-mediated signal transduction by cardiovascular Nox NADPH oxidases. J Mol Cell Cardiol.

[31] Das M, Devi KP, Belwal T, Devkota HP, Tewari D, Sahebnasagh A (2023). Harnessing polyphenol power by targeting eNOS for vascular diseases. Crit Rev Food Sci Nutr.

[32] Prabhu SD, Frangogiannis NG (2016). The biological basis for cardiac repair after myocardial infarction: from inflammation to fibrosis. Circ Res.

[33] Silvis MJM, Kaffka Genaamd Dengler SE, Odille CA, Mishra M, van der Kaaij NP, Doevendans PA (2020). Damage-Associated Molecular Patterns in Myocardial Infarction and Heart Transplantation: The Road to Translational Success. Front Immunol.

[34] Fang Y, Hu J (2011). Toll-like receptor and its roles in myocardial ischemic/reperfusion injury. Med Sci Monit.

[35] Li B, Zhang Q, Du W, Wu J, Cheng J, Zhang Y (2023). Reshaping cardiac microenvironments by macrophage-derived extracellular vesicles-coated Pd@ CeO2 heterostructures for myocardial ischemia/reperfusion injury therapy. Mater Today.

[36] Chen J, Yang J, Liu R, Qiao C, Lu Z, Shi Y (2017). Dual-targeting theranostic system with mimicking apoptosis to promote myocardial infarction repair via modulation of macrophages. Theranostics.

[37] Wang K, Liu CY, Zhou LY, Wang JX, Wang M, Zhao B (2015). APF lncRNA regulates autophagy and myocardial infarction by targeting miR-188-3p. Nat Commun.

[38] Hu D, Li R, Li Y, Wang M, Wang L, Wang S (2024). Inflammation-Targeted Nanomedicines Alleviate Oxidative Stress and Reprogram Macrophages Polarization for Myocardial Infarction Treatment. Adv Sci (Weinh).

[39] Xiang Q, Yi X, Zhu X, Wei X, Jiang D (2023). Regulated cell death in myocardial ischemia-reperfusion injury. Trends Endocrinol Metab.

[40] Christman JW, Blackwell TS, Juurlink BH (2000). Redox regulation of nuclear factor kappa B: therapeutic potential for attenuating inflammatory responses. Brain Pathol.

[41] Guaricci AI, Bulzis G, Pontone G, Scicchitano P, Carbonara R, Rabbat M (2018). Current interpretation of myocardial stunning. Trends Cardiovasc Med.

[42] Zhao R, Jiang S, Zhang L, Yu Z (2019). Mitochondrial electron transport chain, ROS generation and uncoupling. Int J Mol Med.

[43] Neginskaya MA, Pavlov EV, Sheu SS (2021). Electrophysiological properties of the mitochondrial permeability transition pores: Channel diversity and disease implication. Biochim Biophys Acta Bioenerg.

[44] Zaidi A (2010). Plasma membrane Ca2+-ATPases: Targets of oxidative stress in brain aging and neurodegeneration. World J Biol Chem.

[45] Blevins HM, Xu Y, Biby S, Zhang S (2022). The NLRP3 inflammasome pathway: a review of mechanisms and inhibitors for the treatment of inflammatory diseases. Front Aging Neurosci.

[46] Vangheluwe P, Raeymaekers L, Dode L, Wuytack F (2005). Modulating sarco (endo) plasmic reticulum Ca2+ ATPase 2 (SERCA2) activity: cell biological implications. Cell Calcium.

[47] Forgione MA, Cap A, Liao R, Moldovan NI, Eberhardt RT, Lim CC (2002). Heterozygous cellular glutathione peroxidase deficiency in the mouse: abnormalities in vascular and cardiac function and structure. Circulation.

[48] Shen Y, Wang X, Shen X, Wang Y, Wang S, Zhang Y (2022). Geniposide possesses the protective effect on myocardial injury by inhibiting oxidative stress and ferroptosis via activation of the Grsf1/GPx4 axis. Front Pharmacol.

[49] Stocker R, Yamamoto Y, McDonagh AF, Glazer AN, Ames BN (1987). Bilirubin is an antioxidant of possible physiological importance. Science.

[50] Islam MN, Rauf A, Fahad FI, Bin Emran T, Mitra S, Olatunde A (2022). Superoxide dismutase: an updated review on its health benefits and industrial applications. Crit Rev Food Sci Nutr.

[51] Wang T, Zhou T, Xu M, Wang S, Wu A, Zhang M (2022). Platelet membrane-camouflaged nanoparticles carry microRNA inhibitor against myocardial ischaemia-reperfusion injury. J Nanobiotechnology.

[52] Guan Y, Niu H, Wen J, Dang Y, Zayed M, Guan J (2022). Rescuing cardiac cells and improving cardiac function by targeted delivery of oxygen-releasing nanoparticles after or even before acute myocardial infarction. ACS Nano.

[53] Verma DD, Hartner WC, Thakkar V, Levchenko TS, Torchilin VP (2007). Protective effect of coenzyme Q10-loaded liposomes on the myocardium in rabbits with an acute experimental myocardial infarction. Pharm Res.

[54] Yong W, Ma H, Na M, Gao T, Zhang Y, Hao L (2021). Roles of melatonin in the field of reproductive medicine. Biomed Pharmacother.

[55] Mason SA, Trewin AJ, Parker L, Wadley GD (2020). Antioxidant supplements and endurance exercise: current evidence and mechanistic insights. Redox Biol.

[56] Behrendt D, Beltrame J, Hikiti H, Wainstein M, Kinlay S, Selwyn AP (2006). Impact of coronary endothelial function on the progression of cardiac transplant-associated arteriosclerosis: effect of anti-oxidant vitamins C and E. J Heart Lung Transplant.

[57] Caritá AC, Fonseca Santos B, Shultz JD, Michniak Kohn B, Chorilli M, Leonardi GR (2020). Vitamin C: one compound, several uses. Advances for delivery, efficiency and stability. Nanomedicine.

[58] Herbig AL, Renard CM (2017). Factors that impact the stability of vitamin C at intermediate temperatures in a food matrix. Food Chem.

[59] Charlton NC, Mastyugin M, Török B, Török M (2023). Structural features of small molecule antioxidants and strategic modifications to improve potential bioactivity. Molecules.

[60] Cunha Neto F, Marton LT, de Marqui SV, Lima TA, Barbalho SM (2019). Curcuminoids from Curcuma Longa: New adjuvants for the treatment of crohn's disease and ulcerative colitis?. Crit Rev Food Sci Nutr.

[61] Wang Y, Liu X, Chen J, Cao J, Li X, Sun C (2022). Citrus flavonoids and their antioxidant evaluation. Crit Rev Food Sci Nutr.

[62] Liu C, Yao L, Hu Y, Zhao B (2021). Effect of quercetin-loaded mesoporous silica nanoparticles on myocardial ischemia-reperfusion injury in rats and its mechanism. Int J Nanomedicine.

[63] Lozano O, LazaroAlfaro A, SilvaPlatas C, OropezaAlmazan Y, TorresQuintanilla A, BernalRamirez J (2019). Nanoencapsulated quercetin improves cardioprotection during hypoxia-reoxygenation injury through preservation of mitochondrial function. Oxid Med Cell Longev.

[64] Tao T, Liu M, Chen M, Luo Y, Wang C, Xu T (2020). Natural medicine in neuroprotection for ischemic stroke: Challenges and prospective. Pharmacol Ther.

[65] Xu X, Liu A, Hu S, Ares I, Martínez-Larrañaga M-R, Wang X (2021). Synthetic phenolic antioxidants: Metabolism, hazards and mechanism of action. Food Chemistry.

[66] Liu R, Mabury SA (2020). Synthetic phenolic antioxidants: A review of environmental occurrence, fate, human exposure, and toxicity. Environ Sci Technol.

[67] Zhang Z, Dalan R, Hu Z, Wang JW, Chew NW, Poh K (2022). Reactive oxygen species scavenging nanomedicine for the treatment of ischemic heart disease. Adv Mater.

[68] Zhao T, Wu W, Sui L, Huang Q, Nan Y, Liu J (2022). Reactive oxygen species-based nanomaterials for the treatment of myocardial ischemia reperfusion injuries. Bioact Mater.

[69] Qiu M, Singh A, Wang D, Qu J, Swihart M, Zhang H (2019). Biocompatible and biodegradable inorganic nanostructures for nanomedicine: Silicon and black phosphorus. Nano Today.

[70] Majumder J, Taratula O, Minko T (2019). Nanocarrier-based systems for targeted and site specific therapeutic delivery. Adv Drug Deliv Rev.

[71] Lei W, Yang J, Wang J, Xiao Z, Zhou P, Zheng S (2023). Synergetic EGCG and coenzyme Q10 DSPC liposome nanoparticles protect against myocardial infarction. Biomater Sci.

[72] Dvir T, Bauer M, Schroeder A, Tsui JH, Anderson DG, Langer R (2011). Nanoparticles targeting the infarcted heart. Nano Lett.

[73] Shao M, Yang W, Han G (2017). Protective effects on myocardial infarction model: delivery of schisandrin B using matrix metalloproteinase-sensitive peptide-modified, PEGylated lipid nanoparticles. Int J Nanomedicine.

[74] Takahama H, Minamino T, Asanuma H, Fujita M, Asai T, Wakeno M (2009). Prolonged targeting of ischemic/reperfused myocardium by liposomal adenosine augments cardioprotection in rats. J Am Coll Cardiol.

[75] Torchilin VP (2005). Recent advances with liposomes as pharmaceutical carriers. Nat Rev Drug Discov.

[76] Qiu J, Cai G, Liu X, Ma D (2017). αvβ3 integrin receptor specific peptide modified, salvianolic acid B and panax notoginsenoside loaded nanomedicine for the combination therapy of acute myocardial ischemia. Biomed Pharmacother.

[77] Sun L, Hu Y, Mishra A, Sreeharsha N, Moktan JB, Kumar P (2020). Protective role of poly(lactic-co-glycolic) acid nanoparticle loaded with resveratrol against isoproterenol-induced myocardial infarction. BioFactors.

[78] Cheng Y, Liu D, Zhang C, Cui H, Liu M, Zhang B (2019). Mitochondria-targeted antioxidant delivery for precise treatment of myocardial ischemia-reperfusion injury through a multistage continuous targeted strategy. Nanomedicine.

[79] Zhang X, Sun Y, Yang R, Liu B, Liu Y, Yang J (2022). An injectable mitochondria-targeted nanodrug loaded-hydrogel for restoring mitochondrial function and hierarchically attenuating oxidative stress to reduce myocardial ischemia-reperfusion injury. Biomaterials.

[80] Mao S, Wang L, Chen P, Lan Y, Guo R, Zhang M (2018). Nanoparticle-mediated delivery of Tanshinone IIA reduces adverse cardiac remodeling following myocardial infarctions in a mice model: role of NF-κB pathway. Artif Cells Nanomed Biotechnol.

[81] de Paula Peres L, da Luz FAC, dos Anjos Pultz B, Brigido PC, de Araujo RA, Goulart LR (2015). Peptide vaccines in breast cancer: The immunological basis for clinical response. Biotechnol Adv.

[82] Chou LY, Ming K, Chan WC (2011). Strategies for the intracellular delivery of nanoparticles. Chem Soc Rev.

[83] Sun Y, Zhang P, Li Y, Hou Y, Yin C, Wang Z (2022). Light-activated gold-selenium core-shell nanocomposites with NIR-II photoacoustic imaging performances for heart-targeted repair. ACS Nano.

[84] Li H, Zhu J, Xu Y, Mou F, Shan X, Wang Q (2022). Notoginsenoside R1-loaded mesoporous silica nanoparticles targeting the site of injury through inflammatory cells improves heart repair after myocardial infarction. Redox Biol.

[85] Ahmad Shiekh P, Anwar Mohammed S, Gupta S, Das A, Meghwani H, Kumar Maulik S (2022). Oxygen releasing and antioxidant breathing cardiac patch delivering exosomes promotes heart repair after myocardial infarction. Chem Eng J.

[86] Liang Y, Duan L, Lu J, Xia J (2021). Engineering exosomes for targeted drug delivery. Theranostics.

[87] Song H, Chen X, Hao Y, Wang J, Xie Q, Wang X (2022). Nanoengineering facilitating the target mission: targeted extracellular vesicles delivery systems design. J Nanobiotechnology.

[88] Xu M, Feng T, Liu B, Qiu F, Xu Y, Zhao Y (2021). Engineered exosomes: desirable target-tracking characteristics for cerebrovascular and neurodegenerative disease therapies. Theranostics.

[89] Lai J, Pan Q, Chen G, Liu Y, Chen C, Pan Y (2024). Triple hybrid cellular nanovesicles promote cardiac repair after ischemic reperfusion. ACS Nano.

[90] Surman M, Drozdz A, Stepien E, Przybylo M (2019). Extracellular vesicles as drug delivery systems - methods of production and potential therapeutic applications. Curr Pharm Des.

[91] Mu L, Dong R, Guo B (2023). Biomaterials-based cell therapy for myocardial tissue regeneration. Adv Healthc Mater.

[92] Li T, Dong H, Zhang C, Mo R (2018). Cell-based drug delivery systems for biomedical applications. Nano Res.

[93] Luo X, Jiang Y, Li Q, Yu X, Ma T, Cao H (2023). hESC-Derived Epicardial Cells Promote Repair of Infarcted Hearts in Mouse and Swine. Adv Sci (Weinh).

[94] Fang RH, Kroll AV, Gao W, Zhang L (2018). Cell membrane coating nanotechnology. Adv Mater.

[95] Liu Y, Luo J, Chen X, Liu W, Chen T (2019). Cell membrane coating technology: a promising strategy for biomedical applications. Nanomicro Lett.

[96] Yilmaz A, Dengler MA, van der Kuip H, Yildiz H, Rösch S, Klumpp S (2013). Imaging of myocardial infarction using ultrasmall superparamagnetic iron oxide nanoparticles: a human study using a multi-parametric cardiovascular magnetic resonance imaging approach. Eur Heart J.

[97] Li Y, Li B, Wang X, Meng Y, Bai L, Zheng Y (2020). Safe and efficient magnetic resonance imaging of acute myocardial infarction with gadolinium-doped carbon dots. Nanomedicine.

[98] Zheng Y, Zhang H, Hu Y, Bai L, Xue J (2018). MnO nanoparticles with potential application in magnetic resonance imaging and drug delivery for myocardial infarction. Int J Nanomedicine.

[99] Assen Mv, Vonder M, Pelgrim G, Von Knebel Doeberitz P, Vliegenthart R (2020). Computed tomography for myocardial characterization in ischemic heart disease: a state-of-the-art review. Eur Radiol Exp.

[100] Kee PH, Danila D (2018). CT imaging of myocardial scar burden with CNA35-conjugated gold nanoparticles. Nanomedicine.

[101] Choi H, Han JH, Lim SY, Lee I, Cho Y-S, Chun EJ (2017). Imaging of Myocardial Ischemia-Reperfusion Injury Using Sodium [18F] Fluoride Positron Emission Tomography/Computed Tomography in Rats and Humans. Mol Imaging.

[102] Nammas W, Maaniitty T, Knuuti J, Saraste A (2021). Cardiac perfusion by positron emission tomography. Clin Physiol Funct Imaging.

[103] Wei Z, Chen Z, Zhao Y, Fan F, Xiong W, Song S (2021). Mononuclear phagocyte system blockade using extracellular vesicles modified with CD47 on membrane surface for myocardial infarction reperfusion injury treatment. Biomaterials.

[104] Yang L, Ren Y, Pan W, Yu Z, Tong L, Li N (2016). Fluorescent nanocomposite for visualizing cross-talk between microRNA-21 and hydrogen peroxide in ischemia-reperfusion injury in live cells and in vivo. Anal Chem.

[105] Korolev DV, Shulmeyster GA, Istomina MS, Evreinova NV, Aleksandrov IV, Krasichkov AS (2022). Fluorescently Labeled Gadolinium Ferrate/Trigadolinium Pentairon (III) Oxide Nanoparticles: Synthesis, Characterization, In Vivo Biodistribution, and Application for Visualization of Myocardial Ischemia-Reperfusion Injury. Materials.

[106] Zhang Y, Ren X, Wang Y, Chen D, Jiang L, Li X (2021). Targeting ferroptosis by polydopamine nanoparticles protects heart against ischemia/reperfusion injury. ACS Appl Mater Interfaces.

[107] Seshadri G, Sy JC, Brown M, Dikalov S, Yang SC, Murthy N (2010). The delivery of superoxide dismutase encapsulated in polyketal microparticles to rat myocardium and protection from myocardial ischemia-reperfusion injury. Biomaterials.

[108] Zhang S, Wang J, Pan J (2016). Baicalin-loaded PEGylated lipid nanoparticles: characterization, pharmacokinetics, and protective effects on acute myocardial ischemia in rats. Drug Deliv.

[109] Ornatowski W, Lu Q, Yegambaram M, Garcia AE, Zemskov EA, Maltepe E (2020). Complex interplay between autophagy and oxidative stress in the development of pulmonary disease. Redox Biol.

[110] Venardos KM, Kaye DM (2007). Myocardial ischemia-reperfusion injury, antioxidant enzyme systems, and selenium: a review. Curr Med Chem.

[111] Kitagawa S (2014). Metal-organic frameworks (MOFs). Chem Soc Rev.

[112] Guo J, Yang Z, Lu Y, Du C, Cao C, Wang B (2022). An antioxidant system through conjugating superoxide dismutase onto metal-organic framework for cardiac repair. Bioact Mater.

[113] Altshuler PJ, Schiazza AR, Luo L, Helmers MR, Chhay B, Han JJ (2021). Superoxide dismutase-loaded nanoparticles attenuate myocardial ischemia-reperfusion injury and protect against chronic adverse ventricular remodeling. Adv Ther (Weinh).

[114] Berke JD (2018). What does dopamine mean?. Nat Neurosci.

[115] Wu H, Wei M, Xu Y, Li Y, Zhai X, Su P (2022). PDA-based drug delivery nanosystems: a potential approach for glioma treatment. Int J Nanomedicine.

[116] Bao X, Zhao J, Sun J, Hu M, Yang X (2018). Polydopamine nanoparticles as efficient scavengers for reactive oxygen species in periodontal disease. ACS Nano.

[117] Wei Y, Zhu M, Li S, Hong T, Guo X, Li Y (2021). Engineered biomimetic nanoplatform protects the myocardium against ischemia/reperfusion injury by inhibiting pyroptosis. ACS Appl Mater Interfaces.

[118] Cheng W, Zeng X, Chen H, Li Z, Zeng W, Mei L (2019). Versatile polydopamine platforms: synthesis and promising applications for surface modification and advanced nanomedicine. ACS Nano.

[119] Zhou X, McCallum NC, Hu Z, Cao W, Gnanasekaran K, Feng Y (2019). Artificial allomelanin nanoparticles. ACS Nano.

[120] Mo X, Xiang H, Wei L, Xia L, Chen X, Chen Y (2022). Nature-inspired allomelanin nanomedicine alleviates cardiac ischemia/reperfusion injury via scavenging free radicals and ameliorating myocardial microenvironment. Nano Today.

[121] Zhang Y, Yang N, Huang X, Zhu Y, Gao S, Liu Z (2022). Melatonin engineered adipose-derived biomimetic nanovesicles regulate mitochondrial functions and promote myocardial repair in myocardial infarction. Front Cardiovasc Med.

[122] Noh Y, Kim K, Shim M, Choi S, Choi S, Ellisman M (2013). Inhibition of oxidative stress by coenzyme Q10 increases mitochondrial mass and improves bioenergetic function in optic nerve head astrocytes. Cell Death Dis.

[123] Kumar A, Kaur H, Devi P, Mohan V (2009). Role of coenzyme Q10 (CoQ10) in cardiac disease, hypertension and Meniere-like syndrome. Pharmacol Ther.

[124] Rosenfeldt F, Hilton D, Pepe S, Krum H (2003). Systematic review of effect of coenzyme Q10 in physical exercise, hypertension and heart failure. Biofactors.

[125] Li Y, Zhao Y, Yu W, Jiang S (2004). Scavenging ability on ROS of alpha-lipoic acid (ALA). Food Chem.

[126] Gorąca A, HukKolega H, Piechota A, Kleniewska P, Ciejka E, Skibska B (2011). Lipoic acid-biological activity and therapeutic potential. Pharmacol Rep.

[127] Xie D, Zhong Q, Xu X, Li Y, Chen S, Li M (2023). Alpha lipoic acid-loaded electrospun fibrous patch films protect heart in acute myocardial infarction mice by inhibiting oxidative stress. Int J Pharm.

[128] Zhang Y, Zhang Z, Wang R (2020). Protective mechanisms of quercetin against myocardial ischemia reperfusion injury. Front Physiol.

[129] Dong Z, Guo J, Xing X, Zhang X, Du Y, Lu Q (2017). RGD modified and PEGylated lipid nanoparticles loaded with puerarin: formulation, characterization and protective effects on acute myocardial ischemia model. Biomed pharmacother.

[130] Zhang J, Han X, Li X, Luo Y, Zhao H, Yang M (2012). Core-shell hybrid liposomal vesicles loaded with panax notoginsenoside: preparation, characterization and protective effects on global cerebral ischemia/reperfusion injury and acute myocardial ischemia in rats. Int J Nanomed.

[131] Chen M, Wang S, Chen Y, Shen H, Chen L, Ding L (2024). Precision cardiac targeting: empowering curcumin therapy through smart exosome-mediated drug delivery in myocardial infarction. Regen Biomater.

[132] Li L, Wang Y, Guo R, Li S, Ni J, Gao S (2020). Ginsenoside Rg3-loaded, reactive oxygen species-responsive polymeric nanoparticles for alleviating myocardial ischemia-reperfusion injury. J Control Release.

[133] Huang H, Feng W, Chen Y, Shi J (2020). Inorganic nanoparticles in clinical trials and translations. Nano Today.

[134] Kang T, Kim YG, Kim D, Hyeon T (2020). Inorganic nanoparticles with enzyme-mimetic activities for biomedical applications. Coord Chem Rev.

[135] Zhang Y, Yu W, Zhang L, Li P (2024). Nanozyme-based visual diagnosis and therapeutics for myocardial infarction: The application and strategy. J Adv Res.

[136] Liang S, Tian X, Wang C (2022). Nanozymes in the treatment of diseases caused by excessive reactive oxygen specie. J Inflamm Res.

[137] Pansambal S, Oza R, Borgave S, Chauhan A, Bardapurkar P, Vyas S (2023). Bioengineered cerium oxide (CeO2) nanoparticles and their diverse applications: a review. Appl Nanosci.

[138] Ding L, Zhang S, Li Y, Wu Y, Liu X, Xu D (2024). Superoxide dismutase mimetic nanozymes attenuate cardiac microvascular ischemia-reperfusion injury associated with hyperhomocysteinemia. Chem Eng J.

[139] Im GB, Kim YG, Yoo TY, Kim YH, Kim K, Hyun J (2023). Ceria nanoparticles as copper chaperones that activate SOD1 for synergistic antioxidant therapy to treat ischemic vascular diseases. Adv Mater.

[140] Le W, Sun Z, Li T, Cao H, Yang C, Mei T (2024). Antioxidant nanozyme-engineered mesenchymal stem cells for In vivo MRI tracking and synergistic therapy of myocardial infarction. Adv Funct Mater.

[141] Sun Q, Ma H, Zhang J, You B, Gong X, Zhou X (2023). A self-sustaining antioxidant strategy for effective treatment of myocardial infarction. Adv Sci (Weinh).

[142] Ananthakrishnan R, Li Q, O'Shea KM, Quadri N, Wang L, Abuchowski A (2013). Carbon monoxide form of PEGylated hemoglobin protects myocardium against ischemia/reperfusion injury in diabetic and normal mice. Artif Cells Nanomed Biotechnol.

[143] Hofmann R, James SK, Jernberg T, Lindahl B, Erlinge D, Witt N (2017). Oxygen therapy in suspected acute myocardial infarction. N Engl J Med.

[144] Li T, Li J, Liu J, Zhang P, Wu W, Zhou R (2009). Polymerized placenta hemoglobin attenuates ischemia/reperfusion injury and restores the nitroso-redox balance in isolated rat heart. Free Radic Biol Med.

[145] Bice JS, Jones BR, Chamberlain GR, Baxter GF (2016). Nitric oxide treatments as adjuncts to reperfusion in acute myocardial infarction: a systematic review of experimental and clinical studies. Basic Res Cardiol.

[146] Cohen MV, Yang X, Downey JM (2006). Nitric oxide is a preconditioning mimetic and cardioprotectant and is the basis of many available infarct-sparing strategies. Cardiovasc Res.

[147] Heusch G (2015). Molecular basis of cardioprotection: signal transduction in ischemic pre-, post-, and remote conditioning. Circ Res.

[148] Zhu D, Hou J, Qian M, Jin D, Hao T, Pan Y (2021). Nitrate-functionalized patch confers cardioprotection and improves heart repair after myocardial infarction via local nitric oxide delivery. Nat commun.

[149] Xu L, Chen Y, Jin Q, Gao T, Deng C, Wang R (2023). A novel ultrasound-responsive biomimetic nanoparticle for targeted delivery and controlled release of nitric oxide to attenuate myocardial ischemia reperfusion injury. Small Struct.

[150] Sun X, Wang W, Dai J, Jin S, Huang J, Guo C (2017). A long-term and slow-releasing hydrogen sulfide donor protects against myocardial ischemia/reperfusion injury. Sci Rep.

[151] He Y, Zhang B, Chen Y, Jin Q, Wu J, Yan F (2017). Image-guided hydrogen gas delivery for protection from myocardial ischemia-reperfusion injury via microbubbles. ACS Appl Mater Interfaces.

[152] Gui Q, Jiang Z, Zhang L (2021). Insights into the modulatory role of cyclosporine A and its research advances in acute inflammation. Int Immunopharmacol.

[153] Hausenloy DJ, Ong SB, Yellon DM (2009). The mitochondrial permeability transition pore as a target for preconditioning and postconditioning. Basic Res Cardiol.

[154] Li F, Liu D, Liu M, Ji Q, Zhang B, Mei Q (2022). Tregs biomimetic nanoparticle to reprogram inflammatory and redox microenvironment in infarct tissue to treat myocardial ischemia reperfusion injury in mice. J Nanobiotechnology.

[155] Nakamura K, Sata M, Iwata H, Sakai Y, Hirata Y, Kugiyama K (2007). A synthetic small molecule, ONO-1301, enhances endogenous growth factor expression and augments angiogenesis in the ischaemic heart. Clin Sci.

[156] Yajima S, Miyagawa S, Fukushima S, Sakai Y, Iseoka H, Harada A (2019). Prostacyclin analogue-loaded nanoparticles attenuate myocardial ischemia/reperfusion injury in rats. JACC Basic Transl Sci.

[157] Bae S, Park M, Kang C, Dilmen S, Kang TH, Kang DG (2016). Hydrogen Peroxide-Responsive Nanoparticle Reduces Myocardial Ischemia/Reperfusion Injury. J Am Heart Assoc.

[158] Meng W, Zhu J, Wang Y, Shao C, Li X, Lu P (2024). Targeting delivery of miR-146a via IMTP modified milk exosomes exerted cardioprotective effects by inhibiting NF-κB signaling pathway after myocardial ischemia-reperfusion injury. J Nanobiotechnology.

[159] Caldas M, Santos AC, Veiga F, Rebelo R, Reis RL, Correlo VM (2020). Melanin nanoparticles as a promising tool for biomedical applications-a review. Acta Biomater.

[160] Lin Y, Ren J, Qu X (2014). Catalytically active nanomaterials: a promising candidate for artificial enzymes. Acc Chem Res.

[161] Wang L, Qiu S, Li X, Zhang Y, Huo M, Shi J (2023). Myocardial-targeting tannic cerium nanocatalyst attenuates ischemia/reperfusion injury. Angew Chem Int Ed.

[162] Wang X, Guo Z, Ding Z, Mehta JL (2018). Inflammation, autophagy, and apoptosis after myocardial infarction. Journal of the American Heart Association.

[163] Shevtsov MA, Nikolaev BP, Ryzhov VA, Yakovleva LY, Dobrodumov AV, Marchenko YY (2016). Detection of experimental myocardium infarction in rats by MRI using heat shock protein 70 conjugated superparamagnetic iron oxide nanoparticle. Nanomedicine.

[164] Zhu K, Jiang D, Wang K, Zheng D, Zhu Z, Shao F (2022). Conductive nanocomposite hydrogel and mesenchymal stem cells for the treatment of myocardial infarction and non-invasive monitoring via PET/CT. J nanobiotechnology.

[165] Hao J, Lu A, Li X, Li Y (2023). A convergent fabrication of silk fibroin nanoparticles on quercetin loaded metal-organic frameworks for promising nanocarrier of myocardial infraction. Heliyon.

[166] Liao X, Song X, Li J, Li L, Fan X, Qin Q (2022). An injectable co-assembled hydrogel blocks reactive oxygen species and inflammation cycle resisting myocardial ischemia-reperfusion injury. Acta Biomater.

[167] Kang J, Kim H, Mun D, Yun N, Joung B (2021). Co-delivery of curcumin and miRNA-144-3p using heart-targeted extracellular vesicles enhances the therapeutic efficacy for myocardial infarction. J Control Release.

[168] Hardy N, Viola HM, Johnstone VP, Clemons TD, Cserne Szappanos H, Singh R (2015). Nanoparticle-mediated dual delivery of an antioxidant and a peptide against the L-Type Ca2+ channel enables simultaneous reduction of cardiac ischemia-reperfusion injury. ACS Nano.

[169] Tsujioka T, Sasaki D, Takeda A, Harashima H, Yamada Y (2022). Resveratrol-encapsulated mitochondria-targeting liposome enhances mitochondrial respiratory capacity in myocardial cells. Int J Mol Sci.

[170] Zhou H, Shan Y, Tong F, Zhang Y, Tang J, Shen R (2020). Resveratrol nanoparticle complex: potential therapeutic applications in myocardial ischemia reperfusion injury. J Biomed Nanotechnol.

[171] Yang M, Han X, Qiao O (2023). Effect of salvianolic acid B-loaded mesoporous silica nanoparticles on myocardial ischemia-reperfusion injury. Tradit Med Res.

[172] Xiang K, Wu H, Liu Y, Wang S, Li X, Yang B (2023). MOF-derived bimetallic nanozyme to catalyze ROS scavenging for protection of myocardial injury. Theranostics.

[173] Li X, Ren X, Xie M, Zhu M, Zhang Y, Li T (2023). Biominerallized noble metal-based RuO2 nanozymes against myocardial ischemic/reperfusion injury. Adv Nanobiomed Res.

[174] Zhong Y, Yang Y, Xu Y, Qian B, Huang S, Long Q (2024). Design of a Zn-based nanozyme injectable multifunctional hydrogel with ROS scavenging activity for myocardial infarction therapy. Acta Biomater.

[175] Liu W, Zhao N, Yin Q, Zhao X, Guo K, Xian Y (2023). Injectable hydrogels encapsulating dual-functional Au@Pt core-shell nanoparticles regulate infarcted microenvironments and enhance the therapeutic efficacy of stem cells through antioxidant and electrical integration. ACS Nano.

[176] Wang Z, Yang N, Hou Y, Li Y, Yin C, Yang E (2023). L-arginine-loaded gold nanocages ameliorate myocardial ischemia/reperfusion injury by promoting nitric oxide production and maintaining mitochondrial function. Adv Sci (Weinh).

[177] Chen S, Luo X, Sun Y, Jin W, He R (2023). A novel metabolic reprogramming strategy for the treatment of targeting to heart injury-mediated macrophages. Int Immunopharmacol.

[178] Liu X, Chen B, Chen J, Wang X, Dai X, Li Y (2024). A Cardiac-targeted nanozyme interrupts the inflammation-free radical cycle in myocardial infarction. Adv Mater.

[179] Zhang Y, Khalique A, Du X, Gao Z, Wu J, Zhang X (2021). Biomimetic design of mitochondria-targeted hybrid nanozymes as superoxide scavengers. Adv Mater.

[180] Ye T, Chen C, Wang D, Huang C, Yan Z, Chen Y (2024). Protective effects of Pt-N-C single-atom nanozymes against myocardial ischemia-reperfusion injury. Nat Commun.

[181] Li N, Huang C, Zhang J, Zhang J, Huang J, Li S (2023). Chemotactic NO/H2S nanomotors realizing cardiac targeting of G-CSF against myocardial ischemia-reperfusion injury. ACS Nano.

[182] Hao T, Qian M, Zhang Y, Liu Q, Midgley AC, Liu Y (2022). An injectable dual-function hydrogel protects against myocardial ischemia/reperfusion injury by modulating ROS/NO disequilibrium. Adv Sci (Weinh).

[183] Zhang Q, Wang L, Yin Y, Shen J, Xie J, Yuan J (2022). Hydrogen sulfide releasing hydrogel for alleviating cardiac inflammation and protecting against myocardial ischemia-reperfusion injury. J Mater Chem B.

[184] Chen G, Yang L, Zhong L, Kutty S, Wang Y, Cui K (2016). Delivery of hydrogen sulfide by ultrasound targeted microbubble destruction attenuates myocardial ischemia-reperfusion injury. Sci Rep.

[185] Ikeda G, Matoba T, Nakano Y, Nagaoka K, Ishikita A, Nakano K (2016). Nanoparticle-mediated targeting of cyclosporine A enhances cardioprotection against ischemia-reperfusion injury through inhibition of mitochondrial permeability transition pore opening. Sci Rep.

[186] Zhang Z, Chen Z, Yang L, Zhang J, Li Y, Li C (2022). Platelet membrane-encapsulated MSNs loaded with SS31 peptide alleviate myocardial ischemia-reperfusion injury. J Funct Biomater.

[187] Zhang Y, Qian P, Zhou H, Shen R, Hu B, Shen Y (2018). Pharmacological signatures of the exenatide nanoparticles complex against myocardial ischemia reperfusion injury. Kidney Blood Press Res.

[188] Clemons TD, Viola HM, House MJ, Iyer KS, Hool LC (2013). Examining efficacy of “tat-less” delivery of a peptide against the L-type calcium channel in cardiac ischemia-reperfusion injury. ACS Nano.

[189] Zhang M, Zhu J, Qin X, Zhou M, Zhang X, Gao Y (2019). Cardioprotection of tetrahedral DNA nanostructures in myocardial ischemia-reperfusion injury. ACS Appl Mater Interfaces.

